# Diagnosis and treatment of hypertension in dialysis patients: a systematic review

**DOI:** 10.1186/s40885-023-00240-x

**Published:** 2023-09-01

**Authors:** In Soo Kim, Sungmin Kim, Tae-Hyun Yoo, Jwa-Kyung Kim

**Affiliations:** 1 Department of Internal Medicine & Kidney Research Institute, Hallym University Sacred Heart Hospital, Pyungan-dong, Dongan-gu, Anyang, 431-070 Korea; 2 Department of Internal Medicine, College of Medicine, Institute of Kidney Disease Research, Yonsei University, Seoul, Korea

**Keywords:** Hypertension, Hemodialysis, Peritoneal dialysis, Mortality

## Abstract

In patients with end-stage renal disease (ESRD) undergoing dialysis, hypertension is common but often inadequately controlled. The prevalence of hypertension varies widely among studies because of differences in the definition of hypertension and the methods of used to measure blood pressure (BP), i.e., peri-dialysis or ambulatory BP monitoring (ABPM). Recently, ABPM has become the gold standard for diagnosing hypertension in dialysis patients. Home BP monitoring can also be a good alternative to ABPM, emphasizing BP measurement outside the hemodialysis (HD) unit. One thing for sure is pre- and post-dialysis BP measurements should not be used alone to diagnose and manage hypertension in dialysis patients. The exact target of BP and the relationship between BP and all-cause mortality or cause-specific mortality are unclear in this population. Many observational studies with HD cohorts have almost universally reported a U-shaped or even an L-shaped association between BP and all-cause mortality, but most of these data are based on the BP measured in HD units. Some data with ABPM have shown a linear association between BP and mortality even in HD patients, similar to the general population. Supporting this, the results of meta-analysis have shown a clear benefit of BP reduction in HD patients. Therefore, further research is needed to determine the optimal target BP in the dialysis population, and for now, an individualized approach is appropriate, with particular emphasis on avoiding excessively low BP. Maintaining euvolemia is of paramount importance for BP control in dialysis patients. Patient heterogeneity and the lack of comparative evidence preclude the recommendation of one class of medication over another for all patients. Recently, however, β-blockers could be considered as a first-line therapy in dialysis patients, as they can reduce sympathetic overactivity and left ventricular hypertrophy, which contribute to the high incidence of arrhythmias and sudden cardiac death. Several studies with mineralocorticoid receptor antagonists have also reported promising results in reducing mortality in dialysis patients. However, safety issues such as hyperkalemia or hypotension should be further evaluated before their use.

**Supplementary Information:**

The online version contains supplementary material available at 10.1186/s40885-023-00240-x.

## Background

In patients with end-stage renal disease (ESRD) undergoing hemodialysis (HD) or peritoneal dialysis (PD), hypertension is common and often inadequately controlled. Hypertension affects nearly 50–60% of HD patients, although some studies have found that 80–90% of HD patients are affected [1]. It also affects nearly 70–80% of PD patients [2, 3] which is much more common than that in the general population [4]. The prevalence of hypertension in the general Korean adult population aged ≥ 20 years is approximately 30%. The prevalence of hypertension varies widely among studies because of differences in the definition of hypertension and the methods used to measure blood pressure (BP; i.e., before or after dialysis or using ambulatory BP monitoring [ABPM]) [5–7]. And it is well known that dialysis patients have an inverse U- or L-shaped association between BP and risk of death, as opposed to a linear association in the general population. However, when explaining this reverse epidemiology, it is first questioned whether it reflects the accuracy and adequacy of BP measurement in dialysis patients. In addition, the association between BP and mortality may differ between patients on HD and PD, as PD patients are not exposed to the hemodynamic changes associated with HD, such as fluid shifts, intradialytic hypotension, and frequent changes in volume status [8–11]. Here, we discuss the current evidence for the diagnosis and management of hypertension in HD and PD patients.

## HD population

### Accurate measurement of BP in HD patients

The diagnosis and management of hypertension in HD patients is often based on peri-dialysis BP measurements [5]. Peridialytic BP measurements are the BP readings taken by the dialysis unit staff shortly before and after the HD session. This method is widely used for the management of HD patients as well as for epidemiologic studies because of its easy availability in electronic databases of large dialysis units. According to the 2004 National Kidney Foundation Kidney Disease Outcomes Quality Initiative guidelines, hypertension in HD patients is diagnosed when pre-dialysis BP is > 140/90 mmHg or when post-dialysis BP is > 130/80 mmHg [12, 13]. However, peridialytic BP measurements may not fully account for the risk of hypertension and CV events because it is usually measured without the use of the standardized technique [14]. Even when measured according to a standardized protocol, pre- and post-dialysis BP measurements are imprecise estimates of intradialytic or interdialytic BP [15, 16]. Therefore, peri-dialysis BP measurements should not be used alone to diagnose and manage hypertension. Intradialytic BP is a recording measured during HD, typically every 30–60 min, using an automatic cuff attached to the HD machine. The median of intradialytic BP measurements and peridialytic BP recordings may represent an acceptable compromise between utility and practicality when interdialytic BP measurements are not available.

**Fig. 1:**
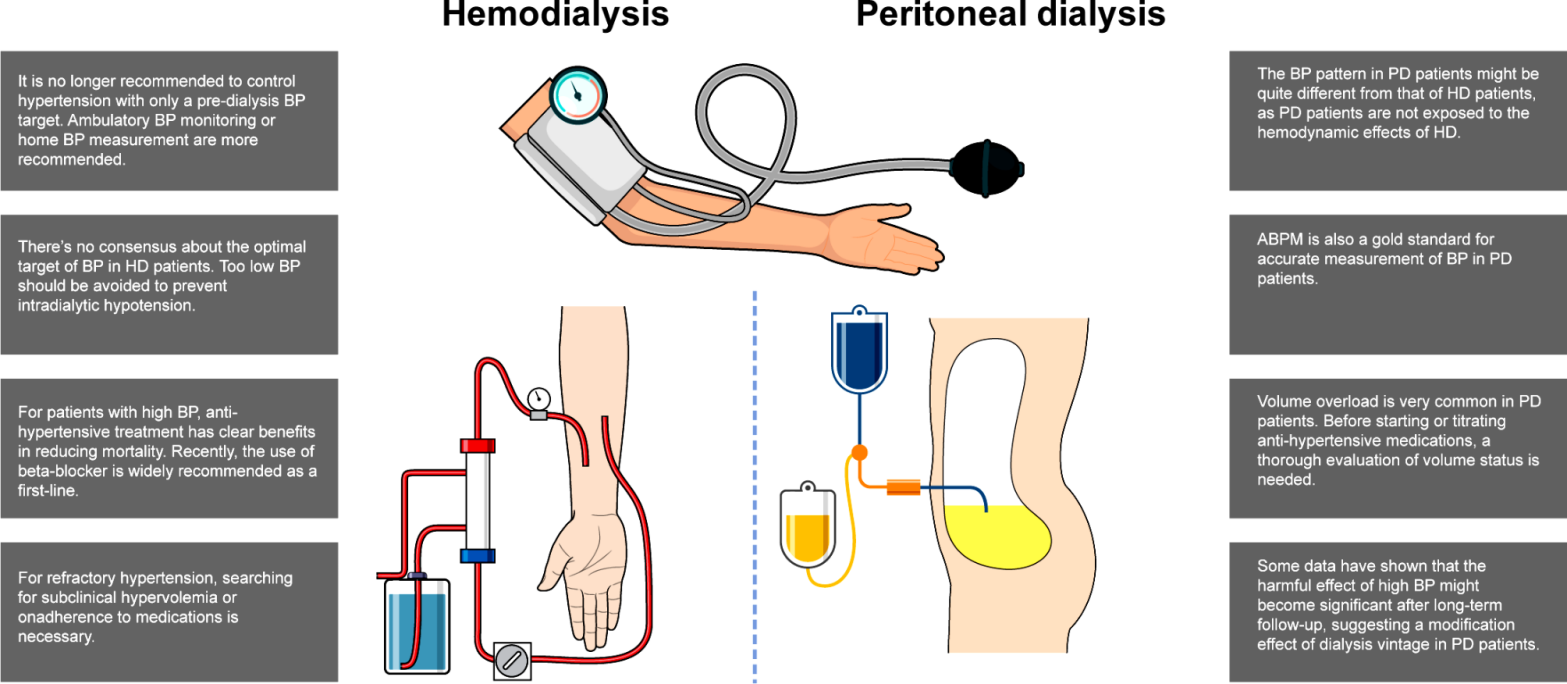
Summary of general management strategy for patients with hemodialysis (HD) or peritoneal dialysis (PD)

Interdialytic BP monitoring is the gold standard for diagnosing hypertension in HD patients (Fig. 1). This can be obtained by ambulatory BP monitoring (ABPM) or by patient self-measurement through home BP monitoring. Regardless of the technique of interdialytic BP assessment, these measurements appear to carry greater prognostic information compared to peridialytic recordings because they provide a more accurate reflection of the patient’s BP load over time [16, 17]. Given the BP variability attributed to interdialytic fluid overload, 44-h ABPM should better delineate cardiovascular morbidity in HD patients. The 44-hour ABPM was initiated at the end of the mid-week dialysis session and continued for 44 h until the next session. (Day 1 was defined as the first 24-hour ABPM and day 2 as the period after day 1 until the next dialysis session). Using the 44 h interdialytic ABPM, hypertension is defined as mean systolic BP (SBP) of ≥ 130 mmHg and/or diastolic BP (DBP) ≥ 80 mmHg or the use of antihypertensive medications [7, 18].

The 44-hour interdialytic ABPM is superior to the peridialytic BP for risk prediction of all-cause and CV mortality [19–21]. If ABPM cannot be performed because of patient intolerance or financial constraints, home BP monitoring is an acceptable alternative. Home BP monitoring can be obtained twice daily in the interdialytic period for 1–2 weeks or twice daily for 4 days after midweek treatment. Compared with peridialytic BP measurement in the HD unit, home BP measurement has a stronger correlation with the mean 44-hour ABPM, higher short-term reproducibility, and better prediction of adverse outcomes [21]. The Chronic Renal Insufficiency Cohort study showed that the SBP measured in the dialysis unit had a U-shaped relationship with mortality, whereas home BP had a linear relationship with all-cause mortality (hazard ratio [HR] = 1.26 for each 10 mmHg increase in SBP; 95% confidence interval [CI] = 1.14–1.40), similar to the general population [20]. The key disadvantages of home BP monitoring are the inability to assess nocturnal dipping and high cost. Another alternative to the use of ABPM is in-office BP measurement outside of the dialysis unit. Increased SBP outside of the dialysis unit is an independent risk factor for mortality [22, 23].

Another important issue that complicates the accurate diagnosis of hypertension is the high BP variability in HD patients. In HD patients, BP varies over the very short-term (beat-to-beat), short-term (within 24 h), mid-term (day-to-day), and long-term (visit-to-visit). BP variability mainly depends on the volume status and arterial stiffness and is associated with target-organ damage and mortality [24]. However, it is unclear whether BP variability is a modifiable risk factor for mortality in HD patients. Therefore, studies of interventions targeting BP variability are required [24].

### BP and risk for CV events and death in HD patients

The relationship between BP and all-cause mortality or cause-specific mortality in HD patients is unclear [25, 26]. Previous observational studies of the HD cohort have nearly universally reported a U-shaped or even an L-shaped association between BP and all-cause mortality, with much higher risk at low BP and either no or only a small increase in mortality at high BP [27, 28]. One of the largest prospective observational cohorts of chronic HD patients in the French Observatory, including 9,333 individuals, showed the lowest HR of all-cause mortality with a pre-dialysis SBP of 165 mmHg [28]. In this study, the 95% lower CI was approximately 135/70 mmHg, indicating more harm with low BP than with high BP. Unfortunately, there is only one relevant pilot randomized controlled trial (RCT) comparing the CV benefits of different BP targets in the HD population, the Blood Pressure in Dialysis (BID) pilot study. In this study, 126 participants were randomized to an intensive pre-dialysis SBP goal of 110–140 mmHg or a standard SBP goal of 155–165 mmHg [29]. At 12 months, a mean difference in SBP of 12.9 mmHg was achieved; however, there were no significant differences in changes in the left ventricular mass (LVM) between the intensive and standard goal groups (median difference = − 0.84 g/m^2^, interquartile range [IQR] = − 17.1 to 10.0 and median difference = 1.4 g/m^2^, IQR = − 11.6 to 10.4, respectively; p = 0.43). However, an insignificant increase in the risk of hospitalization and vascular access thrombosis was observed in the intensive arm compared with the standard arm, suggesting non-intensive goals for pre-dialysis SBP in HD patients [29]. Other studies have also reported that reducing pre-dialysis SBP may increase the incidence of intradialytic hypotension [30, 31], major adverse CV events [32, 33], and vascular access thrombosis [34]. Although some studies have suggested that these detrimental effects of low BP are primarily related to non-cardiac causes, such as poor physiological reserve and frailty due to comorbid conditions [35], all of these data raised substantial concerns about whether lowering BP overall is a strategy for lowering mortality in HD patients [28, 36–41].

Nevertheless, a meta-analysis of RCTs in HD patients showed a significant benefit of BP reduction with antihypertensive treatment on CV events and CV mortality. A 2009 systematic review and meta-analysis of eight RCTs and 1,679 HD patients found that BP reduction with antihypertensive treatment was associated with a 29% decreased risk of CV events (relative risk [RR], 0.71, 95% CI 0.55–0.92, p = 0.009), a 20% decreased risk for all-cause mortality (RR, 0.80, 95% CI, 0.66–0.96, p = 0.014), and a 29% decreased risk of CV mortality (RR, 0.71, 95% CI, 0.50–0.99, p = 0.044) [42], emphasizing the need for routine BP reduction in individuals undergoing dialysis. Similarly, another meta-analysis published in 2009, which included 5 RCTs and 1,202 HD patients, showed that compared with placebo or control treatment, BP reduction with antihypertensive treatment resulted in a 31% reduction in the risk of CV events using a fixed-effects model and by 38% using a random-effects model [43]. All the included studies had HRs for CV events of 0.29–0.93 [40, 44–47]. In addition, CV protective effect with anti-hypertensive treatment was observed in both hypertensive and normotensive patients with LV systolic dysfunction. Then, it is necessary to correctly interpret the results of the previous pilot study, BID data, which showed no difference (statistically insignificant) in the incidence rates of major adverse CV events, hospitalizations, and vascular access thrombosis between the intensive arm and standard arm. Although some researchers interpret the study results as favoring non-intensive treatment of pre-dialysis SBP, however, it can also be interpreted as meaning that intensive BP lowering did not worsen the incidence of these outcomes. This means that it may be a possible safety signal. Therefore, a comprehensive, large-scale RCT is required to assess the potential benefits of intensive BP control in HD patients.

This discrepancy may be partly explained by the inadequacy of peridialytic BP recordings per se to describe the true BP load. In fact, prospective cohort studies have shown that interdialytic BP recorded either at home or by ABPM is clearly more associated with mortality, whereas the association between peridialytic BP recordings and all-cause and CV mortality was unclear [48]. A previous study found that self-measured home SBP of 125 to 145 mmHg and ambulatory BP of 115 to 125 mmHg were associated with the best prognosis in 150 HD patients [20]. In the largest study to date, conducted in 326 mainly African American patients, those in the higher quartiles of home and 44-hour ambulatory SBP had an excess risk of mortality was independent of other risk factors over 32 months of follow-up [19].

In addition, it has been suggested that elevated pulse pressure (PP)/arterial stiffness and/or comorbidities may be more important determinants of future outcomes than specific BP cut-off levels in dialysis patients. For example, an analysis of 24,525 patients from the DOPPS study showed that the U-shape between BP and mortality was mostly observed for SBP (pre-dialysis SBP < 130 mmHg or > 160 mmHg was associated with higher mortality), but not for DBP, where a higher mortality rate was only observed in patients with pre-dialysis DBP < 60 mmHg, suggesting that increased PP/arterial stiffness may be responsible for these associations [37]. Similarly, post-dialysis PP have been shown to be associated with an increased risk of death, suggesting that increased PP may be a causal factor in cardiovascular disease [49, 50]

Lastly, some studies have shown time-varying effects of BP on outcomes, emphasizing that dialysis duration may modify the association between BP and mortality. Stidley et al.reported that pre-dialysis SBP < 120 mmHg was associated with increased mortality in the first 2 years and that adverse effects of high SBP were only apparent after 3 years of follow-up [51]. Mazzuchi et al. also found an association between low DBP and early mortality and between high SBP and late mortality in 405 HD patients [35].

In summary, the impact of BP reduction on HD patients is still unclear. Based on the above data, accurate BP measurement is one of the most important. In addition, well-designed RCTs are needed to determine the long-term effects of BP lowering on patient outcomes.

### Therapeutic target of optimal BP

Based on the SPRINT study [52], the updated 2021 Kidney Disease: Improving Global Outcomes (KDIGO) BP guidelines strongly recommend lowering SBP to < 120 mmHg (standardized office BP) in patients with CKD, if tolerated, but there is less certainty about the ideal BP in HD patients [53, 54]. Although some very outdated guidelines, including the 2005 Kidney Disease Outcomes Quality Initiative (K/DOQI)^13^, the 2006 HD guideline of the Canadian Society of Nephrology [55], and the 2012 guideline of the Japanese Society for Dialysis Therapy [56] suggest a pre-dialysis BP target of < 140/90 mmHg, recent guidelines have not mentioned optimal BP targets. As a result, an individualized approach to BP management is appropriate for HD patients, with a particular focus on avoiding hypotension. In addition, attention should be paid to intradialytic and interdialytic BP patterns, volume management, and comorbidities.

One thing for sure is that it is no longer recommended to control hypertension with only a pre-dialysis BP target. Instead, an interdialytic self-measured home BP or use of mean/median peridialytic BPs are recommended as mentioned above [20]. Based on the results of several data, an average home BP ≥ 135/85 mmHg or ambulatory BP ≥ 130/80 mmHg is considered hypertension, and in general, the target for self-measured home BP in HD patients is less than 130/80 mmHg in HD patients [19]. If interdialytic self-measured home BP is not available, targeting a median midweek BP of < 140/80 mmHg appears to be a reasonable alternative strategy. The median midweek BP can be calculated from all BPs measured during a midweek dialysis session (e.g., on Wednesday for a patient receiving HD on Mondays, Wednesdays, and Fridays).

### Intradialytic hypotension and hypertension

In a typical HD session, BP decreases from pre-dialysis to post-dialysis; the magnitude of this decrease is closely related to the ultrafiltration (UF) volume [16]. Intradialytic hypotension is a serious complication of HD and is associated with vascular access thrombosis, inadequate dialysis dose, and mortality [34, 57]. It is one of the reasons to be cautious about lowering pre-dialysis SBP too much before HD. The prevalence of intradialytic hypotension ranges from 15 to 50% depending on the definition [30]. Overall, an absolute nadir SBP of < 90 mmHg is most significantly associated with mortality. Therefore, a symptomatic decrease in BP or a nadir intradialytic SBP of < 90 mmHg should prompt a reassessment of BP management [30]. This reassessment includes, but is not limited to, an evaluation of UF rate, dialysis treatment time, interdialytic weight gain, dry weight (DW) estimation, and antihypertensive medication use. However, avoidance of intradialytic hypotension should not be at the expense of maintaining euvolemia or ensuring adequate dialysis time.

Intradialytic hypertension is characterized by a paradoxical increase in BP during or immediately after a dialysis session, when most of the excess fluid has already been removed. Its pathogenesis is unclear, although some evidence suggests that activation of the sympathetic nervous system and renin-angiotensin system (RAS), endothelial stiffness, volume excess, and other mechanisms may be involved. Intradialytic hypertension occurs in 5–15% of patients. Previous observational data have demonstrated that each 10 mmHg increase in SBP during HD is independently associated with a 6% increase in the HR for death [58]. Furthermore, these findings are most pronounced in patients with a pre-dialysis SBP of < 120 mmHg. Although the exact mechanism of this relationship is unclear, studies have suggested that intradialytic hypertension is associated with volume excess and interdialytic hypertension [59, 60]. Therefore, an increase in SBP of > 10 mmHg from pre- to post-dialysis into the hypertensive range should prompt a detailed evaluation of the interdialytic BP pattern and volume management, including out-of-unit BP measurements and a critical assessment of the DW. Table 1 summarizes information for the diagnosis of hypertension in HD patients.

**Table 1:** Summary of diagnosis of hypertension in HD patients

• ABPM is the gold standard for the diagnosing hypertension in HD patients. If ABPM is not available, home BP recordings can be a good alternative for accurate BP measurements.
• An average home BP ≥ 135/85 mmHg or ambulatory BP ≥ 130/80 mmHg is considered high BP in HD patients, and in general, the target of self-measured home BP is less than 130/80 mmHg.
• If neither ABPM nor home BP measurements are available, in-office BP measurements outside of the dialysis unit or median midweek peridialytic BP may be acceptable.
• Increased pulse pressure/arterial stiffness may be another determinant in predicting adverse CV outcomes.
• There may be a time-varying effect between high BP and mortality, suggesting the need for long-term follow-up data to predict mortality.
• Intradialytic BP pattern should also be considered to avoid serious complications of HD.

## PD population

To date, most studies of optimal BP targets in dialysis patients have largely been conducted in HD patients; data from PD patients are very limited. The main difference between PD and HD is that PD is a continuous, machine-free dialysis method performed at home. With continuous nature (dialysis for 24 h), PD is generally thought to preserve residual renal function better than HD and does not commonly induce intradialytic hypotension. With these advantages, PD can more easily control volume status, so there are fewer dietary and fluid restrictions for PD patients than for HD patients. However, fluid overload (FO) is thought to be more common in PD than in HD patients, largely due to less fluid restriction. To date, epidemiologic data have shown similar results (i.e., high BP is associated with increased death rates) between HD and PD, but because PD patients are not exposed to the hemodynamic effects of HD and experience higher rates of subclinical hypervolemia, there may be some difference in the relationship with mortality [10, 61].

In the European Body Composition Monitoring study, bioimpedance analysis (BIA) revealed that only 40% of 639 PD patients were euvolemic [62]. Similarly in the Initiative of Patient Outcomes in Dialysis study, BIA showed subclinical overhydration in 57% of 1,092 PD patients [63]. A reduction in extracellular water has been reported to be associated with regression of LV mass index (LVMI) [64]. Therefore, the first approach to hypertension in PD patients should always be to evaluate and optimize volume status (Fig. 1). In this regard, preservation of residual renal function and peritoneal membrane function should be taken care by minimizing dialysate glucose exposure, appropriate use of icodextrin, salt restriction, and adequate diuretic use [63]. The detailed PD prescription to maintain euvolemic status is not described as it is beyond the scope of this review.

The 2015 International Society of Peritoneal Dialysis guideline suggests a target BP of < 140/90 mmHg in PD patients [65], but a review of current evidence raises some controversial issues regarding this suggestion. First, ABPM is also the gold standard for accurate BP measurement in PD patients, as it is in HD patients and the general population. However, PD data assessing the validity of peridialytic, office, and home BP or the associations between out-of-unit BP measurements and the risk of CV death are limited. In particular, volume-mediated changes in the ambulatory BP rhythms (i.e., interdialytic high BP caused by weight gain in HD patients) are thought to be less pronounced in PD patients, because of the “steady” volume state.

However, a recent comparative study between HD and PD showed that dialysis modality did not affect ABPM during any of the periods studied [66]. Very similar to HD patients, previous PD data have reported the importance of arterial stiffness and increased PP on mortality [67, 68]. as well as the excess risk of low BP on mortality in PD patients, too [69, 70]. However, the effect of high SBP may vary over time in PD patients, suggesting a modifying effect of dialysis vintage. In a cohort of 2,770 PD patients, Udayaraj et al. have shown that greater SBP was associated with decreased mortality in the first year, but was associated with increased late mortality (in years 6+).^61^ These findings suggest the importance of long-term follow-up to determine the effect of BP on mortality in PD patients.

In general, similar to HD patients, an average home BP ≥ 135/85 mmHg or ambulatory BP ≥ 130/80 mmHg is considered high BP in PD patients. Based on the advantages of PD as home dialysis, further research is needed to standardize home BP measurement and to provide optimal target values. Table 2 summarized the information for the diagnosis of hypertension in HD patients.

**Table 2:** Summary of diagnosis of hypertension in PD patients

• Chronic clinical or subclinical hypervolemia is very common in PD patients. In PD patients with high BP, an assessment of volume status should be a priority.
• ABPM is the gold standard for the diagnosis of hypertension in PD patients. However, data assessing the validity of peridialytic, office, and home BP are limited in PD patients.
• A mean home BP ≥ 135/85 mmHg or ambulatory BP ≥ 130/80 mmHg is also considered high BP in PD patients.
• Similar to the HD patients, there may be an effect of dialysis vintage on the relationship between high BP and long-term mortality in PD patients.

## Treatment of hypertension in dialysis patients

### Non-pharmacological intervention for volume control

In dialysis patients with hypertension, non-pharmacological treatments, including a reduced target DW, should be considered because volume overload underlies most cases of BP elevation in HD and PD patients [71]. DW is defined as the lowest-tolerated post-dialysis body weight, achieved through a gentle and gradual reduction of post-dialysis weight, at which patients experience minimal signs or symptoms of hypovolemia or hypervolemia [72]. If appropriate, the target DW should be adjusted before antihypertensive agents are added, because gradual DW reduction can normalize the BP or make BP control easier [73–76]. Even in patients with normal pre-dialysis BP, those with high post-dialysis BP show increased extracellular water content, suggesting volume overload [73]. Several assessment tools have been developed to evaluate the volume status of patients. A discussion of the methods used to measure the extracellular water content and strategies to reduce the DW during dialysis is beyond the scope of this review.

### Minimization of inter- and intra-dialytic sodium gain

Because of the minimal or absent sodium and fluid excretory capacity of ESRD patients, their BP is typically salt-sensitive [77]. Salt and fluid restriction are the cornerstone of non-pharmacological strategies for volume management; however, evidence regarding their effectiveness are surprisingly scarce. Dietary sodium restriction effectively controls thirst, reduces interdialytic weight gain, and facilitates the achievement of optimal DW and BP control [78]. Although the serum level of sodium that triggers thirst varies across individuals, most patients maintain their pre-dialysis sodium levels within the normal range. These findings suggest that water intake is adjusted to match salt intake, which highlights the importance of emphasizing salt restriction, rather than the overly simplistic advice to only restrict fluid intake. In fact, fluid restriction without concomitant sodium restriction is not supported by the evidence and is often not feasible due to increased thirst [79]. Therefore, dietary sodium intake in dialysis patients should not exceed 65 mmol (1.5 g sodium or 4 g sodium chloride). In patients with low pre-dialysis sodium levels, other issues such as poorly controlled glucose levels or excessive water intake should be considered.

In general, PD patients should follow the aforementioned recommendations for sodium restriction. The modification of PD regimens with low-sodium or icodextrin solutions may facilitate sodium and volume control. A nonrandomized interventional study compared the use of a standard PD solution and low-sodium PD solution during a single 3–5 h exchange per day over a mean follow-up period of 2 months. The use of the low-sodium dialysate resulted in a significant increase in diffusive peritoneal sodium removal of 30–50 mmol/dwell, which was accompanied by reduced thirst, lower total body water, and a decrease in nighttime SBP by 8 mmHg [80].

### Pharmacologic approaches

If the BP remains above the target despite non-pharmacological measures for volume control, initiation or up-titration of antihypertensive medications is necessary. If BP is well-controlled but antihypertensive medications interfere with the UF (e.g., by causing intradialytic hypotension), the medication dose may be reduced to enhance the UF. When antihypertensive medications are already being used for BP control and cardio-protection, it is reasonable to continue them unless they interfere with achieving the DW target. It is difficult to determine whether the benefits of antihypertensive drugs used in HD patients are because of their BP-lowering effects or other non-hemodynamic effects, because previous studies have not appropriately evaluated the ambulatory or home BP. Patient heterogeneity and scarcity of comparative evidence precludes recommending any medication class over the other for all patients [81].

#### Angiotensin-converting enzyme inhibitors (ACEis)/angiotensin receptor blockers (ARBs)

RAS blockers are the first-line antihypertensive medications in the general population and may also be appropriate for hypertensive patients receiving dialysis [82]. Most ARBs are not dialyzed during conventional dialysis and may be preferred for sustained BP reduction in dialysis patients. However, RCTs have not confirmed that RAS blockade offers similar benefits in dialysis patients as in the general population. In the Fosinopril in Dialysis Trial conducted in 2006, 397 HD patients were randomized to receive the ACEI fosinopril or placebo for a mean follow-up period of 48 months. The participants had left ventricular hypertrophy (LVH) but were not necessarily hypertensive. Although treatment with fosinopril resulted in a significant reduction of pre-dialysis BP compared to placebo, the occurrence of fatal and nonfatal CV events did not significantly differ between the two groups [45]. Another phase III RCT conducted in Italy revealed that the use of ramipril (titrated to the maximally tolerated dose) did not reduce the risk for major CV events; however, hypotensive episodes were more common in the ramipril group than in the control [83]. Peters et al. also failed to show a benefit of irbesartan on biomarkers of arterial stiffness, LVM, and autonomic nerve function in HD patients [84]. The largest study conducted to date is the Olmesartan Clinical Trial in Okinawa Patients under Dialysis Study, which was conducted in Japan. A total of 469 hypertensive HD patients were randomly assigned to olmesartan (10–40 mg per day) or another treatment that does not include ARBs and ACEis and followed up for 3.5 years. Compared with patients receiving other medications, olmesartan treatment was not associated with a significant reduction in BP (mean difference in BP = 0.9 mmHg) or in the incidence of fatal and nonfatal CV events (HR = 1.00, 95% CI = 0.62–1.52) [85]. Based on these data, two meta-analyses including 837 and 900 HD patients, reported no significant reduction in fatal and nonfatal CV events in patients treated with ACEis or ARBs compared with those in a standard care group [86, 87]. To date, no study has demonstrated superiority of ACEis or ARBs over other antihypertensive meidcations in dialysis patients, and anti-hypertensive treatment, rather than the use of an RAS blocker, seems to be the factor associated with a reduced CV risk.

#### β-blockers

Some studies have suggested that β-blockers should be used as the first-line antihypertensive treatment (Fig. 1) [88]. The rationale for their use is that sympathetic overactivity in dialysis patients significantly predicts the risk of premature death and CV events [89]. Sympathetic overactivity can partly explain the high incidence of arrhythmias and sudden cardiac death in dialysis patients. Therefore, β-blockers are an attractive treatment option for CV protection [90]. Furthermore, more than 70% of HD patients have LVH at baseline and 30% have coronary artery disease at the initiation of dialysis. Several studies have reported the superiority of β-blockers over other antihypertensive treatments in preventing sudden death, reducing the all-cause mortality rate, and improving the LV function [44, 91]. With 200 maintenance HD patients with echocardiographic LVH and hypertension, Agarwal et al. performed an RCT comparing the efficacy of reducing LVMI (primary outcome) between lisinopril and atenolol (the HDPAL trial). At baseline, the 44-hour ambulatory BP was similar between both groups, but at 12 months, atenolol led to a numerically greater reduction of BP according to the 44-hour interdialytic ABPM (mean reduction = − 21/–13 vs. − 18/–10 mmHg, respectively) and self-measured home BP readings (mean reduction = − 25/–12 vs. − 19/–10 mmHg, respectively) compared to lisinopril. More importantly, this trial was terminated early due to the superiority of atenolol over lisinopril for the prevention of adverse CV outcomes. The rate of the combined outcome of myocardial infarction, stroke, and hospitalization for heart failure or CV death was 2.29-fold higher with lisinopril-based treatment than with atenolol-based treatment (incidence rate ratio = 2.29; 95% CI = 1.07–5.21).^88^ The dose-limiting side effect of β-blockers is bradycardia. Among patients with symptomatic bradycardia from β-blockers (e.g., lightheadedness, presyncope or syncope, exercise intolerance), in such cases, the dose should be reduced.

The dialyzability of β-blockers should be considered [92]. Some β-blockers are efficiently removed from the circulation by HD (i.e., high dialyzability; atenolol, acebutolol, and metoprolol), whereas others are not (i.e., low dialyzability; carvedilol and propranolol). This characteristic may influence the efficacy of β-blockers in HD patients, possibly due to the preserved intradialytic protection against arrhythmias. In general, the use of non-dialyzable β-blockers is advisable, as a propensity-matched retrospective cohort study suggested that there may be no survival benefit from by highly dialyzable β-blockers in dialysis patients. However, evidence on the effect of drug dialyzability is scarce. A recent prospective cohort study of 15,699 HD patients from Taiwan showed that the use of dialyzable β-blockers was associated with lower all-cause mortality compared to the use of non-dialyzable β-blockers [93]. Another systemic review also reported higher mortality rates with the use of the non-dialyzable carvedilol compared to the highly dialyzable metoprolol, which was attributed to a higher likelihood of intradialytic hypotension with carvedilol [94]. Therefore, drug dialyzability may affect intradialytic BP changes, and it may be prudent to avoid non-dialyzable medications in cases of frequent intradialytic hypotension. For relatively stable intradialytic BP, the use of longer-acting, once-daily medications may improve adherence and reduce pill burden. It is reasonable to select medications based on patient characteristics, cardiovascular indications, and availability.

#### Calcium channel blockers (CCB)

Dihydropyridine CCBs are potent antihypertensive agents that effectively lower the BP, even in the volume-overloaded state [95]. These drugs are often safely used for the management of hypertension in dialysis patients. However, few RCTs have evaluated the outcomes of CCB use. Small studies have suggested that dihydropyridine CCBs are equally effective as ACEis or ARBs for reducing LVH and carotid intima-media thickness [96]. Evidence on the use of non-dihydropyridine CCBs in HD patients is scarce, and their use in HD patients should follow the recommendations for the general population. Notably, all CCBs are not removed during standard HD and their pharmacokinetics are unchanged in ESRD [97].

#### Mineralocorticoid receptor antagonists (MRA)

Mineralocorticoid receptor antagonists, such as spironolactone, are commonly used in non-dialysis patients with resistant hypertension. In general, their use is avoided in HD patients because of the potential risk of hyperkalemia. However, two recent trials have reported promising results with spironolactone in dialysis patient. In the Dialysis Outcomes Heart Failure Aldactone Study [98], 309 oligoanuric HD patients were randomized to receive 25 mg/day of spironolactone without any restriction of dietary potassium intake (treatment group), and 152 patients were assigned to a control group. During the 3-year follow-up, spironolactone significantly reduced the risks of death from CV events or hospitalization before (HR = 0.40, 95% CI = 0.20–0.81) and after adjustment (HR = 0.38, 95% CI = 0.17–0.83), respectively. The incidence of drug discontinuation due to serious hyperkalemia was 1.9%. Another multicenter RCT randomized 253 HD or PD patients without heart failure to 2 years of spironolactone (25 mg/day) or placebo. Add-on MRA therapy reduced the occurrence of the composite primary end point of CV mortality and mitigated the risks for cardiac arrest and sudden death (HR = 0.42, 95% CI = 0.26–0.78), suggesting beneficial effects of low-dose spironolactone on reducing CV morbidity and mortality in dialysis patients [99]. Importantly, the two aforementioned studies suggest that the mortality reduction with spironolactone exceeds 50% in dialysis patients, which is surprising because few studies have shown such a significant reduction of the mortality rate of dialysis patients [100]. Indeed, a cardioprotective effect of MRAs in dialysis patients has an established biological basis [101, 102]. The beneficial effect of MRAs is mediated through improved endothelial function and reduced LV size independent of BP changes, rather than through changes in salt or potassium handling by the kidney. However, the safety of MRAs in this population should be evaluated further [102]. In addition, which mineralocorticoid receptor antagonist is most suitable for use in ESRD is still questionable. Newer agents, such as finerenone, may have a better safety profile, although this needs further study. The ongoing study Aldosterone Antagonist Chronic Hemodialysis Interventional Survival Trial (ALCHEMIST; NCT01848639) is expected to determine the effectiveness and safety of MRAs in the ESRD patients. Table 3 summarized information for treatment of hypertension in HD patients.

**Table 3:** Summary of treatment of hypertension in dialysis patients

• Patient heterogeneity and lack of comparative evidence preclude the recommendation of one class of drug over another for all dialysis patients.
• Most ARBs are not dialyzed during conventional dialysis and can be used for sustained BP reduction. However, RCTs have failed to confirm the benefit of RASi in dialysis patients as in the general population.
• β-blockers may be used as the first-line therapy in dialysis patients because they can control the sympathetic overactivity and LVH which contribute to the high incidence of arrhythmias and sudden cardiac death.
• In a recent study, the use of mineralocorticoid receptor antagonists such as spironolactone showed promising results in reducing mortality by more than 50% in dialysis patients. However, safety issues such as hyperkalemia or hypotension should be further evaluated.
• Volume overload or nonadherence to medications are common causes of resistant hypertension in dialysis patients.

## Resistant hypertension

Resistant hypertension is defined as uncontrolled hypertension despite the use of at least three drugs of different classes including diuretics or hypertension controlled with at least four drugs. According to data from the Korean Ambulatory Blood Pressure Monitoring Registry, the prevalence of resistant hypertension in the general population is about 12% [103]. In the dialysis population, the prevalence is much higher. European multicenter data with 506 HD patients showed that the prevalence of resistant hypertension with 44-hour ABPM criteria (≥ 130/80 mmHg) was estimated at 25% [104]. Although fluid overload is a central feature of resistant hypertension in HD patients [105], in that study, fluid overload per se explains the 33% of resistant hypertension and the 67% of patients showed no fluid overload. Non-adherence to medications is another common cause of resistant hypertension [106]. Chronically non-adherent hypertensive patients who refuse to take medications at home may benefit from the administration of long-acting antihypertensive medications in the dialysis unit. If a treatable cause cannot be found, minoxidil may be effective in reducing BP. The central sympathetic agonists, such as methyldopa and clonidine, are used less frequently because of their adverse effects involving the central nervous system [107, 108].

Finally, recent RCTs have confirmed the ability of renal denervation to lower BP in patients that are resistant to the BP-lowering effects of multiple antihypertensive drugs. Evidence is limited, however, in patients with ESRD. Renal denervation is an experimental therapy in which sympathetic nerves innervating the kidney are ablated for BP control. The effect of renal denervation was evaluated in a small nonrandomized trial of 24 HD patients who showed resistant hypertension despite maximal medical therapy with confirmed adherence [109]. The baseline office and 24-hour mean SBP in the renal denervation group were 180 ± 112 and 175 ± 11 mmHg, respectively. After renal denervation, an early and persistent reduction of SBP was observed (office SBP: 165 ± 13; 150 ± 7 and 149 ± 11 mmHg; 24-hr SBP 163 ± 20, 148 ± 10 and 149 ± 17 mmHg after 1, 6 and 12 months, respectively). The BP-lowering effect was almost always present and statistically significant during both the day and night, suggesting the beneficial role of renal denervation also in dialysis patients.

## Conclusions

Hypertension is very common in the dialysis population, but the diagnosis of hypertension and the optimal treatment target are unclear. At present, however, it is clear that high BP is associated with increased CV events and that the use of anti-hypertensive medications is beneficial in reducing mortality in dialysis patients. Interdialytic BP monitoring, ABPM or home BP monitoring, is superior to the traditional peridialytic BP measurements for predicting long-term outcomes. For treatment of high BP, dietary sodium restriction and maintenance of euvolemic status are of paramount importance. Overall, all anti-hypertensive drugs can be used in dialysis population, with more recent recommendations for the use of β-blocker as first-line therapy. RCTs with anti-hypertensive drugs selection aimed at reducing mortality are still needed.

### Electronic supplementary material

Below is the link to the electronic supplementary material.

Supplementary Material 1

## Data Availability

Not applicable.

## List of abbreviations

ABPM: Ambulatory blood pressure monitoring
ACEi: Angiotensin-converting enzyme inhibitor
ARB: Angiotensin receptor blocker
BIA: Bioimpedance analysis
BID: Blood Pressure in Dialysis
BP: Blood pressure
CCB: Calcium channel blockers
CI: Confidence interval
CV: Cardiovascular
DBP: Diastolic blood pressure
DW: Dry weight
ESRD: End-stage renal disease
HD: Hemodialysis
HR: Hazard ratio
IQR: interquartile range
KDIGO: Kidney Disease: Improving Global Outcomes
K/DOQI: Kidney Disease Outcomes Quality Initiative
LV: Left ventricle
LVM: Left ventricular mass
LVMI: Left ventricular mass index
MACE: Major adverse cardiovascular event
MRA: Mineralocorticoid receptor antagonist
PD: peritoneal dialysis
PP: pulse pressure
RAS: Renin-angiotensin system
RCT: Randomized controlled trial
RR: Relative risk
SBP: Systolic blood pressure
UF: ultrafiltration
