# Supplementary material for: Diagnosis and treatment of hypertension in dialysis patients: a systematic review
Source: Clin Hypertens. 2023 Sep 1;29:24. doi: 10.1186/s40885-023-00240-x (PMC10472689; doi:10.1186/s40885-023-00240-x)
Supplement: Supplementary file 1 — Supplementary Material 1 [file 40885_2023_240_MOESM1_ESM.docx]

**Diagnosis and treatment of hypertension in dialysis patients: A systematic review**

In Soo Kim^1^, Sungmin Kim^1^, Tae-Hyun Yoo, MD, PhD^2^, Jwa-Kyung Kim, MD, PhD^1^

^1^Department of Internal Medicine, Hallym University Sacred Heart Hospital, Anyang, Korea, ^2^ Department of Internal Medicine, College of Medicine, Institute of Kidney Disease Research, Yonsei University, Seoul, Korea

**Corresponding Author**

**Jwa Kyung Kim, MD, PhD**

Department of Internal Medicine & Kidney Research Institute,

Hallym University Sacred Heart Hospital,

Pyungan-dong, Dongan-gu, Anyang, Korea, 431-070

Tel: 82-31-380-3720

E-mail: kjk816@hallym.or.kr

**Abstract**

In patients with end-stage renal disease (ESRD) undergoing dialysis, hypertension is common but often inadequately controlled. The prevalence of hypertension varies widely among studies because of differences in the definition of hypertension and methods of measuring blood pressure (BP; i.e., peri-dialysis or using ambulatory BP recordings). Recently, ambulatory blood pressure monitoring (ABPM) is regarded as the gold standard for the diagnosis of hypertension in dialysis patients. Also, home BP recordings can be a good alternative for ABPM, emphasizing the measurement BP outside the hemodialysis (HD) unit. One thing for sure is pre- and post-dialysis BP measurements should not be used alone to diagnose and manage hypertension. The exact target of BP as well as the relationship between BP and all-cause mortality or cause-specific mortality are unclear in dialysis population. A lot of observational studies with HD cohort have nearly universally noted a U-shaped or even an L-shaped association between BP and all-cause mortality, however most of these data are based on the BP measured in HD unit. Some data with ABPM have shown a linear association between BP and mortality even in HD patients, similar to the general population. Supporting this, the results of meta-analysis have demonstrated a clear benefit of BP reduction in HD patients. So, further research for targeting optimal BP is needed in dialysis population, and for now, an individualized approach for BP management is appropriate with a particular focus on avoiding an excessively low BP. Maintaining an euvolemic state is of utmost importance to control BP in dialysis patients. Patient heterogeneity and scarcity of comparative evidence preclude recommending any medication class over the other for all patients. However, recently, β-blockers might be considered as the first-line therapy in dialysis patients since they can control the sympathetic overactivity and left ventricular hypertrophy, which contribute to the high incidence of arrhythmias and sudden cardiac death. Also, several trials with mineralocorticoid receptor antagonists reported promising results in reducing mortality in dialysis patients. Safety issues such as hyperkalemia or hypotension should be further evaluated before using them, though.

**Keywords**

Hypertension, hemodialysis, peritoneal dialysis, mortality

**Background**

In patients with end-stage renal disease (ESRD) undergoing hemodialysis (HD) or peritoneal dialysis (PD), hypertension is common and often inadequately controlled. Hypertension affects almost 50–60% of HD patients, although some studies have found that 80–90% of HD patients are affected.^1^ Furthermore, it affects almost 70–80% of PD patients^2, 3^ which is much more common than that in the general population.^4^ The prevalence of hypertension in the general Korean adult population aged ≥ 20 years is approximately 30%. The prevalence of hypertension varies widely among studies because of differences in the definition of hypertension and methods of measuring blood pressure (BP; i.e., before or after dialysis or using ambulatory BP recordings).^5-7^ Dialysis patients are known to have an inverse U- or L-shaped association between BP and risk of death, as opposed to a linear association in the general population. However, when explaining this reverse epidemiology, it is first questioned whether the accuracy and adequacy of BP measurement in dialysis patients are reflected. Limited evidence is available on the diagnosis and management of hypertension in patients on HD and PD, although cardiovascular (CV) complications are the leading cause of mortality in this population. Furthermore, the association between BP and mortality may differ between patients on PD and HD, as PD patients are not exposed to the hemodynamic changes associated with HD, such as fluid shift, intradialytic hypotension, and frequent change in volume status.^8-11^ Herein, we discuss the current evidence for the diagnosis and treatment of hypertension in HD and PD patients.

**HD population**

***1) Accurate measurement of BP in HD patients***

The diagnosis and management of hypertension in HD patients is often based on peri-dialysis BP measurements.^5^ Peridialytic BP measurements are the BP readings taken by the dialysis unit staff shortly before and after the HD session. This method is widely used for the management of HD patients as well as for epidemiologic studies because of its easy availability in electronic databases of large dialysis units. According to the 2004 National Kidney Foundation Kidney Disease Outcomes Quality Initiative guidelines, hypertension in HD patients is diagnosed when pre-dialysis BP is >140/90 mmHg or when post-dialysis BP is >130/80 mmHg.^12, 13^ However, these BP measurements may not fully account for the risks for hypertension and CV events because peridialytic BP measurements are usually recorded by the dialysis unit staff without the use of the standardized technique.^14^ Even when measured according to a standardized protocol, pre- and post-dialysis BP measurements are imprecise estimates of interdialytic BP.^15^ Several observational studies have shown that pre- and post-dialysis BP measurements do not correlate with those recorded outside the dialysis unit.^16^ Therefore, pre- and post-dialysis BP measurements should not be used alone to diagnose and manage hypertension. Intradialytic BP is a recording measured during HD, typically every 30-60 minutes, using an automatic cuff attached to the HD machine. The median of intradialytic BP measurements and peridialytic BP recordings may represent an acceptable compromise between utility and practicality when interdialytic BP measurements are not available.

Recently, the importance of accurate BP measurement, particularly outside the dialysis unit, has been emphasized. Ambulatory BP monitoring (ABPM) provides accurate BP measurements and is the gold standard for the diagnosis of hypertension in patients with chronic kidney disease who are receiving dialysis. (Fig.1)^16, 18^ Given the BP variability attributed to interdialytic fluid overload, 44-h ABPM should better delineate cardiovascular morbidity in HD patients. The 44-hour ABPM was initiated at the end of the mid-week dialysis session and continued for 44 hours until the next session. (Day 1 was defined as the first 24-hour ABPM and day 2 as the period after day 1 until the next dialysis session). Using the 44 h interdialytic ABPM, hypertension is defined as mean systolic BP (SBP) of ≥130 mmHg and/or diastolic BP (DBP) ≥ 80 mmHg or the use of antihypertensive medications.^7, 19^ The 44 h interdialytic ABPM is superior to the peridialytic BP for risk prediction of all-cause and CV mortality.^20-22^ The Chronic Renal Insufficiency Cohort study showed that the SBP measured in the dialysis unit had a U-shaped relationship with mortality, whereas home BP had a linear association with all-cause mortality (hazard ratio [HR] = 1.26 for each 10 mmHg increase in SBP; 95% confidence interval [CI] = 1.14–1.40), similar to the general population.^21^ If ABPM cannot be performed because of patient intolerance or financial constraints, home BP recordings are an acceptable alternative. Home BP recordings can be obtained two times a day in the interdialytic period over 1–2 weeks or two times a day for 4 days following the midweek treatment. Compared to the peridialytic BP measurement in HD unit, home BP measurement has a stronger correlation with the mean 44 h ABPM, higher short-term reproducibility, and better prediction of adverse outcomes.^22^ The key disadvantages of home BP monitoring are inability to evaluate for nocturnal dipping and high cost. Another alternative to the use of ABPM is in-office BP measurement outside of the dialysis unit. Increased SBP outside of the dialysis unit is an independent risk factor for mortality.^23^ Finally, mean or median peridialytic BP has higher sensitivity and specificity for the detection of interdialytic hypertension compared to pre- and post-dialysis BP measurements alone.^24^ However, no studies have assessed the association of this approach with patient outcomes.

Another important issue that complicates the accurate diagnosis of hypertension is the high BP variability in HD patients. In HD patients, BP varies over the very short-term (beat-to-beat), short-term (within 24 hours), mid-term (day-to-day), and long-term (visit-to-visit). BP variability mainly depends on the volume status and arterial stiffness and is associated with target-organ damage and mortality.^17^ However, it is unclear whether BP variability is a modifiable risk factor for mortality in HD patients. Therefore, studies of interventions targeting BP variability are required.^17^

***2) BP and risk for CV events and death in HD patients***

The relationship between BP and all-cause mortality or cause-specific mortality in HD patients is unclear.^25, 26^ Previous observational studies with HD cohort have nearly universally noted a U-shaped or even an L-shaped association between BP and all-cause mortality, with much higher risk at low BP and either no or only mild increase in mortality wit high BP.^27, 28^ One of the largest observational prospective French Observatory cohorts of chronic HD patients including 9,333 individuals showed the lowest HR of all-cause mortality with a pre-dialysis SBP of 165 mmHg.^28^ In that study. the 95% lower CI was approximately 135/70 mmHg, indicating more harm with low BP than with high BP. Unfortunately, so far, there is only one relevant pilot randomized controlled trial (RCT) comparing the CV benefits of different BP target in the HD population, the Blood Pressure in Dialysis (BID) pilot study. In this study, 126 participants were randomly assigned to an intensive pre-dialysis SBP goal of 110–140 mm Hg or a standard SBP goal of 155–165 mmHg.^29^ At 12 months, a mean difference in SBP of 12.9 mmHg was achieved; however, there were no significant differences in changes in the left ventricular mass (LVM) between the intensive and standard goal groups (median difference = –0.84 g/m^2^, interquartile range [IQR] = –17.1 to 10.0 and median difference = 1.4 g/m^2^, IQR = –11.6 to 10.4, respectively; p = 0.43). However, a insignificant increase in the risks of hospitalization and vascular access thrombosis were observed in the intensive arm compared to the standard arm, suggesting non-intensive goals of pre-dialysis SBP in HD patients.^29^ Other studies also have reported that reducing pre-dialysis SBP may increase the frequency of intradialytic hypotension^30, 31^, major adverse CV events^32, 33^, and vascular access thrombosis^34^. Although some studies suggested that these detrimental effects of low BP are associated with primarily non-cardiac origin such as poor physiological reserve and frailty due to comorbid conditions,^35^ however, all these data raised substantial concerns about whether lowering BP, as a whole, is a strategy for lowering mortality in the HD patients.^28, 36-41^

Nevertheless, a meta-analysis of RCTs of HD patients demonstrated a significant benefit of BP reduction with antihypertensive treatment on CV events and CV mortality. A 2009 systematic review and meta-analysis of eight RCTs and 1,679 HD patients found that BP reduction with antihypertensive treatment was associated with a 29% decreased risk for CV events, 20% decreased risk for all-cause mortality, and 29% decrease risk for CV mortality (RR, 0.71, 95% CI, 0.50-0.99, p = 0.044)^42^, emphasizing the need for routine BP reduction in individuals undergoing dialysis to reduce the high risk for CV morbidities and mortality. Similarly, another meta-analysis published in 2009 which included 5 RCTs and 1,202 HD patients showed that compared to placebo or control treatment, BP reduction with antihypertensive treatment resulted in a 31% reduction in risk for CV events (pooled HR, 0.69, 95% CI, 0.56-0.84) using a fixed-effects model and by 38% using a random-effects model.^43^ The included studies had HRs for CV events of 0.29–0.93, while none of the studies suggested harm with non-intensive treatment of pre-dialysis SBP in HD patients.^40, 44-47^ In addition, CV protective effect with anti-hypertensive treatment was observed both in hypertensive and normotensive patients with left ventricular systolic dysfunction. Then, it is needed to correctly interpret the results of the previous pilot study, BID data, showing no difference (statistically insignificant) in the incidence rate ratios of major adverse cardiac events (MACE), hospitalizations, and vascular access thrombosis between the intensive arm and standard arm. Although some researchers interpret the study results as favoring non-intensive treatment of pre-dialysis SBP, however, it can also be interpreted as intensive BP lowering did not aggravate the incidence of those outcomes. This means that it may be a possible safety signal. Therefore, a comprehensive, large-scale RCT is required to assess the potential benefits of intensive BP control in HD patients.

These findings can partly be explained by the inadequacy of peridialytic BP recordings per se to describe the true BP load. In fact, prospective cohort studies have shown that interdialytic BP recorded either at home or by ABPM is clearly more associated with mortality and CV events, while the association between peridialytic BP recordings and all-cause and CV mortality was unclear. A study on 57 hypertensive HD patients who prospectively followed for a mean period of 34.4 ± 20.4 months reported that elevated 24 h ambulatory BP as well as elevated nocturnal SBP, were independently associated with increased risk for CV mortality.^48^ Similarly a larger study found that self-measured home SBP of 125 to 145 mmHg and of 115 to 125 mmHg by ambulatory BP were associated with the best prognosis in 150 HD patients.^21^ In the largest study performed to date, undertaken in 326 mainly African American patients, those in the higher quartiles of home and 44 h ambulatory SBP exhibited an excessive risk for mortality, which was independent of other risk factors over 32 months of follow-up.^20^

In addition, some researchers have suggested that increased pulse pressure (PP)/arterial stiffness and/or comorbid conditions are more important determinants for CV outcomes, rather than some cut-off levels of BP. For example, an analysis of 24,525 patients from the DOPPS study indicated that the U-shape between BP and mortality was mostly observed for SBP (pre-dialysis SBP < 130 mmHg or >160 mmHg was associated with higher mortality), but not for DBP, where a higher mortality rate was only observed in patients with pre-dialysis DBP <60 mmHg, suggesting that increased PP/arterial stiffness may be responsible for these associations.^37^ Some previous studies have shown that post-dialysis PP is associated with an increased risk of death, suggesting that increased PP may be a causal factor in cardiovascular disease ^49, 50^

Other studies have revealed time-varying effects of BP on outcomes, emphasizing that dialysis may modify the association between BP and mortality. Stidley *et al.*reported that pre-dialysis SBP < 120 mmHg was associated with increased mortality in the first 2 years and that adverse effects of high SBP were apparent only beyond 3 years of follow-up in HD patients.^51^ Mazzuchi *et al.* also found a associations between low DBP and early mortality and between high SBP and late mortality in 405 HD patients.^35^

In summary, the effects of BP reduction on HD patients are unclear. Based on the aforementioned data, accurate BP measurement is important. Furthermore, it is more important to determine the long-term effects of BP reduction on patient outcomes rather than examining the extent of BP reduction.

***3) Therapeutic target of optimal BP***

Based on the SPRINT trial^52^, the updated 2021 Kidney Disease: Improving Global Outcomes (KDIGO) BP guidelines strongly recommend lowering SBP to <120 mmHg in chronic kidney disease patients (standardized office BP), when tolerated, but there is less certainty regarding the ideal BP in dialysis patients.^53, 54^ Based on the existing evidence, definitive recommendations regarding the BP targets for dialysis patients cannot be made. The results of the pilot BID study and several epidemiological studies suggest that a too low pre-dialysis SBP target may be associated with an increased CV risk. Although some very outdated guidelines, including the 2005 Kidney Disease Outcomes Quality Initiative (K/DOQI)^13^, the 2006 HD guideline from the Canadian Society of Nephrology^55^, and the 2012 guideline from the Japanese Society for Dialysis Therapy^56^ suggest a pre-dialysis BP target of <140/90 mmHg in HD patients, recent guidelines have not mentioned optimal BP targets. As a result, an individualized approach for BP management is appropriate for patients receiving dialysis, with a particular focus on avoiding an excessively low BP. Furthermore, attention should be paid to intradialytic and interdialytic BP patterns, volume management, and comorbidities.

One thing for sure is that it is no longer recommended to control hypertension with only a pre-dialysis BP target. Instead, an interdialytic self-measured home BP or use of mean/median peridialytic BPs are recommended as mentioned above.^21^ Based on the results of several data, an average home BP ≥ 135/85 mmHg or ambulatory BP ≥ 130/80 mmHg is considered hypertension, and in general, the target for self-measured home BP in HD patients is less than 130/80 mmHg in HD patients.^20^ If interdialytic self-measured home BP is not available, targeting a median midweek BP of <140/80 mmHg appears to be a reasonable alternative strategy. The median midweek BP can be calculated from all BPs measured during a midweek dialysis session (e.g., on Wednesday for a patient receiving HD on Mondays, Wednesdays, and Fridays).

***4) Intradialytic hypotension and hypertension***

In a typical HD session, BP decreases from pre- to post-dialysis; the magnitude of this reduction is closely related to the ultrafiltration (UF) volume.^16^ Intradialytic hypotension is a serious complication of HD and is associated with vascular access thrombosis, inadequate dialysis dose, and mortality.^34, 57^ It is one of the reasons to be careful not to lower pre-dialysis SBP too much before HD. The prevalence of intradialytic hypotension ranges from 15% to 50% depending on the definition.^30^ Overall, an absolute nadir SBP of <90 mmHg is most significantly associated with mortality. Therefore, symptomatic decreases in BP or a nadir intradialytic SBP of < 90 mmHg should prompt a reassessment of BP management.^30^ This reassessment includes, but is not limited to, an evaluation of UF rate, dialysis treatment time, interdialytic weight gain, dry weight (DW) estimation, and antihypertensive medication use. However, the avoidance of intradialytic hypotension should not come at the expense of maintaining euvolemia or ensuring adequate dialysis time.

Intradialytic hypertension is characterized by paradoxical BP elevation during or immediately after a dialysis session, when most of the excess fluid has already been removed. Its pathogenesis is unclear, although some evidence suggests that activation of the sympathetic nervous system and renin-angiotensin system (RAS), endothelial stiffness, volume excess, and other mechanisms may be involved. Intradialytic hypertension affects 5–15% of patients. Previous observational data of HD patients have demonstrated that every 10 mmHg increase in SBP during HD is independently associated with a 6% increase in the HR of death.^58^ Moreover, these findings are most prominent in patients with a pre-dialysis SBP of <120 mmHg. Although the exact mechanism of this relationship is unclear, studies have suggested that intradialytic hypertension is associated with volume excess and interdialytic hypertension..^59, 60^ Therefore, an increase in SBP of >10 mmHg from pre- to post-dialysis into the hypertensive range should prompt a detailed evaluation of the inter-dialytic BP pattern and volume management, including out-of-unit BP measurements and a critical assessment of the DW. Table 1 summarized information for diagnosis of hypertension in HD patients.

**PD population**

To date, most studies of optimal BP targets in dialysis patients have largely been conducted in HD patients; data from PD patients are very limited. The main difference between PD and HD is that PD is a continuous, machine-free dialysis method performed at home. With continuous nature (dialysis for 24 hours), PD is generally thought to preserve residual renal function better than HD and does not commonly induce intradialytic hypotension. With these advantages, PD can more easily control volume status, so there are fewer dietary and fluid restrictions for PD patients than for HD patients. However, fluid overload (FO) is thought to be more common in PD than in HD patients, largely due to less fluid restriction. To date, epidemiologic data have shown similar results (i.e., high BP is associated with increased death rates) between HD and PD, but because PD patients are not exposed to the hemodynamic effects of HD and experience higher rates of subclinical hypervolemia, there may be some difference in the relationship with mortality.^10, 61^

In the European Body Composition Monitoring study, bioimpedance analysis (BIA) uncovered that only 40% of 639 PD patients were euvolemic.^62^ Similarly in the Initiative of Patient Outcomes in Dialysis study, BIA uncovered subclinical overhydration in 57% of 1,092 PD patients.^63^ A reduction in extracellular water was reported to be associated with regression of LV mass index (LVMI).^64^ Therefore, the first approach to hypertension in PD patients should always be evaluation and optimization of volume status (Fig.1). In this regard, preservation of residual renal function and peritoneal membrane function should be taken care by minimizing dialysate glucose exposure, appropriate use of icodextrin, salt restriction, and diuretic usage.^63^ The detailed PD prescription to maintain euvolemic status will not be described because it is out of the scope of this review.

The 2015 International Society of Peritoneal Dialysis guideline suggests a target BP of <140/90 mmHg in PD patients^65^, but reviewing current evidence raises some controversial issues regarding this suggestion. First, for accurate measurement of BP, ambulatory BP monitoring is also the gold standard in PD patients, as it is in HD patients and the general population. However, data assessing the validity of peridialytic, office, and home BP or the associations between out-of-unit BP measurements and the risk for CV death are limited. Just the volume-mediated changes in the ambulatory BP rhythms (i.e., interdialytic weight gain-associated interdialytic high BP in HD patients) is thought to be less prominent in PD patients, owing to the “steady” volume state.

However, a recent comparative study between HD and PD showed that dialysis modality did not affect ABPM during any of the periods studied.^66^ Very similar to HD patients, previous PD data have reported the importance of arterial stiffness and increased PP on mortality.^67, 68^ as well as the excess risk of low BP on mortality in PD patients, too.^69, 70^ However, the effect of high SBP may vary over time in PD patients, suggesting a modifying effect of dialysis vintage. In a cohort of 2,770 PD patients, Udayaraj *et al.* have shown that greater SBP was associated with decreased mortality in the first year, but was associated with increased late mortality (in years 6+).^61^ These findings suggest the importance of long-term follow-up to determine the effect of BP on mortality in PD patients.

Previous cohort studies with PD patients showed the excess risk with low BP on mortality.^69, 70^ However, the effect of high SBP might vary over time in PD patients, suggesting a modification effect of dialysis vintage. In a cohort of 2,770 PD patients, Udayaraj *et al.* have shown that greater SBP, DBP, and PP were associated with decreased mortality in the first year, but greater SBP and PP were associated with increased late mortality (in years 6+).^61^ These findings suggest the importance of long-term follow-up to determine the effect of BP on mortality in PD patients.

Similar to HD patients, an average home BP ≥ 135/85 mmHg or ambulatory BP ≥ 130/80 mmHg is regarded as high BP in PD patients, too. Based on the PD’s merits as a home-based dialysis, further research to standardizing home BP measurement in PD patients should be studied in the future. Table 2 summarized information for diagnosis of hypertension in HD patients.

**Hypertension treatment in dialysis patients**

***1) Non-pharmacological intervention for volume control***

For dialysis patients with hypertension, non-pharmacological treatments, including a reduced DW target, should be considered, as volume overload underlies most cases of BP elevation in HD and PD patients.^71^ DW is defined as the lowest-tolerated post-dialysis body weight, achieved through a gentle and gradual reduction of post-dialysis weight, at which patients experience minimal signs or symptoms of hypovolemia or hypervolemia.^72^ If appropriate, the target DW should be adjusted before antihypertensive agents are added, because gradual DW reduction can normalize the BP or make BP control easier.^73-76^ Even in patients with normal pre-dialysis BP, those with high post-dialysis BP show increased extracellular water content, suggesting volume overload..^73^ Several assessment tools have been developed to evaluate the volume status of patients. A discussion of the methods used to measure the extracellular water content and strategies to reduce the DW during dialysis is out of the scope of this review.

***2) Minimization of inter- and intra-dialytic sodium gain***

Because of the minimal or absent sodium and fluid excretory capacity of ESRD patients, their BP is typically salt-sensitive.^77^ Salt and fluid restriction are the cornerstone of non-pharmacological strategies for volume management; however, evidence regarding their effectiveness are surprisingly scarce. Dietary sodium restriction effectively controls thirst, reduces interdialytic weight gain, and facilitates the achievement of optimal DW and BP control.^78^ Although the serum level of sodium that triggers thirst varies across individuals, most patients maintain their pre-dialysis sodium levels within the normal range. These findings suggest that water intake is adjusted to match salt intake, which highlights the importance of emphasizing salt restriction, rather than the overly simplistic advice to only restrict fluid intake. Indeed, fluid restriction without concomitant sodium restriction is not supported by evidence and is frequently not feasible due to increased thirst.^79^ Thus, in dialysis patients, dietary sodium intake should not exceed 65 mmol (1.5 g sodium or 4 g sodium chloride). For patients with low pre-dialysis sodium levels, other issues should be considered, such as poorly controlled glucose levels or excessive water intake. Dietary interventions to reduce interdialytic weight gain should be administered cautiously to avoid negative effects on nutritional status, particularly in frail patients.

In general, PD patients should follow the aforementioned recommendations for sodium intake restriction. The modification of PD regimens with low-sodium or icodextrin solutions may facilitate sodium and volume control. A nonrandomized interventional study compared the use of a standard PD solution and low-sodium PD solution during a single 3–5 h exchange per day over a mean follow-up period of 2 months. The use of the low-sodium dialysate resulted in a significant increase in diffusive peritoneal sodium removal of 30–50 mmol/dwell, which was accompanied by reduced thirst, lower total body water, and a decrease in nighttime SBP by 8 mmHg.^80^

***3) Pharmacologic approaches***

If the BP remains above the target despite non-pharmacological measures for volume control, initiation or up-titration of antihypertensive medications is necessary. If BP is well-controlled but antihypertensive medications interfere with the UF (e.g., by causing intradialytic hypotension), the medication dose may be reduced to enhance the UF. When antihypertensive medications are already being used for BP control and cardio-protection, it is reasonable to continue them unless they interfere with achieving the DW target. It is difficult to determine whether the benefits of antihypertensive drugs used in HD patients are because of their BP-lowering effects or other non-hemodynamic effects, because previous studies have not appropriately evaluated the ambulatory or home BP. Patient heterogeneity and scarcity of comparative evidence precludes recommending any medication class over the other for all patients.^81^

1) Angiotensin-converting enzyme inhibitors (ACEis)/angiotensin receptor blockers (ARBs)

RAS blockers are the first-line antihypertensive medications for the general population and may also be appropriate for hypertensive patients receiving dialysis.^82^ Most ARBs are not dialyzed during conventional dialysis and may be preferred for sustained BP reduction in dialysis patients. However, RCTs have not confirmed that RAS blockade offers similar benefits in dialysis patients as in the general population. In the Fosinopril in Dialysis Trial conducted in 2006, 397 HD patients were randomized to receive the ACEI fosinopril or placebo for a mean follow-up period of 48 months. The participants had left ventricular hypertrophy (LVH) but were not necessarily hypertensive. Although treatment with fosinopril resulted in a significant reduction of pre-dialysis BP compared to placebo, the occurrence of fatal and nonfatal CV events did not significantly differ between the two groups.^45^ Another phase III RCT conducted in Italy revealed that the use of ramipril (titrated to the maximally tolerated dose) did not reduce the risk for major CV events; however, hypotensive episodes were more common in participants allocated to a ramipril group than in control.^83^ Peters *et al*. also failed to show a benefit of irbesartan on biomarkers of arterial stiffness, LVM, and autonomic nerve function in HD patients.^84^ The largest study conducted to date is the Olmesartan Clinical Trial in Okinawa Patients under Dialysis Study, which was conducted in Japan. A total of 469 hypertensive HD patients were randomly assigned to olmesartan (10–40 mg per day) or or another treatment that does not include ARBs and ACEis and followed up for 3.5 years. Compared to patients who received other medications, olmesartan treatment was not associated with a significant reduction in BP (mean difference in BP = 0.9 mmHg) or incidence of fatal and nonfatal CV events (HR = 1.00, 95% CI = 0.62–1.52).^85^ Based on these data, two meta-analyses, including 837 and 900 HD patients, reported no significant reduction in fatal and nonfatal CV events among patients treated with ACEis or ARBs compared to those in a standard care group.^86, 87^ To date, no study has demonstrated superiority of ACEis or ARBs over other antihypertensive drugs in dialysis patients, and anti-hypertensive treatment, rather than the use of an RAS blocker, seems to be the factor associated with a reduced CV risk.

2) β-blockers

Some studies have suggested that β-blockers should be used as the first-line antihypertensive treatment (Fig.1).^88^ The rationale for their use is that sympathetic overactivity in dialysis patients significantly predicts the risks of premature death and CV events.^89^ The sympathetic overactivity can partly explain the high incidence of arrhythmias and sudden cardiac death in dialysis patients. Therefore, β-blockers are an attractive treatment option for CV protection.^90^ Furthermore, more than 70% of HD patients have LVH at baseline and 30% have coronary artery disease at the initiation of dialysis. Several studies have reported the superiority of β-blockers over other antihypertensive treatments in preventing sudden death, reducing the all-cause mortality rate, and improving the left ventricular function.^44, 91^ With 200 maintenance HD patients with echocardiographic LVH and hypertension, Agarwal *et al.* performed an RCT comparing the efficacy of reducing LVMI (primary outcome) between lisinopril and atenolol (the HDPAL trial). At baseline, the 44 h ambulatory BP was similar between both groups, but at 12 months, atenolol led to a numerically greater reduction of BP according to the 44 h interdialytic ABPM (mean reduction = –21/–13 vs. –18/–10 mmHg, respectively) and self-measured home BP readings (mean reduction = –25/–12 vs. –19/–10 mmHg, respectively) compared to lisinopril. More importantly, this trial was terminated early due to the superiority of atenolol over lisinopril for the prevention of adverse CV outcomes. The rate of the combined outcome of myocardial infarction, stroke, and hospitalization for heart failure or CV death was 2.29-fold higher with lisinopril-based treatment than atenolol-based treatment (incidence rate ratio = 2.29; 95% CI = 1.07–5.21).^88^ The dose-limiting side effect of β-blockers is bradycardia. Among patients with symptomatic bradycardia from β-blockers (eg, lightheadedness, presyncope or syncope, exercise intolerance), in such cases, the dose should be reduced.

The dialyzability of β-blockers should be considered when prescribing them for dialysis patients^92^. Some β-blockers are efficiently removed from the circulation by HD (i.e., high dialyzability; atenolol, acebutolol, and metoprolol), whereas others are not (i.e., low dialyzability; carvedilol and propranolol). This characteristic may influence the effectiveness of β-blockers in HD patients, possibly due to the preserved intradialytic protection against arrhythmias. In general, the use of non-dialyzable β-blockers is advisable, because a propensity-matched retrospective cohort study suggested that a survival benefit may not be offered by highly dialyzable β-blockers in dialysis patients. However, evidence regarding the effects of drug dialyzability is scarce. A recent prospective cohort study of 15,699 HD patients from Taiwan showed that the use of dialyzable β-blockers was associated with lower all-cause mortality compared to the use of non-dialyzable β-blockers.^93^ Another systemic review also reported higher mortality rates with the use of the non-dialyzable carvedilol compared to the highly dialyzable metoprolol, which was attributed to a higher likelihood of intradialytic hypotension with carvedilol.^94^ Therefore, drug dialyzability may affect intradialytic BP changes, and it may be prudent to avoid non-dialyzable medications in cases of frequent intradialytic hypotension. For relatively stable intradialytic BP, the use of longer-acting, once-daily medication may improve adherence and reduce pill burden. It is reasonable to select medications based on patient characteristics, cardiovascular indications, and availability.

3) Calcium channel blockers (CCB)

Dihydropyridine CCBs are potent antihypertensive agents that effectively lower the BP, even in the volume-expanded state.^95^ These drugs are often safely used for the management of hypertension in dialysis patients. However, few RCTs have evaluated the outcomes of CCB use. Small studies have suggested that dihydropyridine CCBs are equally effective as ACEis or ARBs for reducing LVH and carotid intima-media thickness.^96^ Evidence on the use of non-dihydropyridine CCBs in HD patients is scarce, and their use in HD patients should follow the recommendations for the general population. Notably, all CCBs are not removed during standard HD and their pharmacokinetics are unchanged in ESRD.^97^

4) Mineralocorticoid receptor antagonists (MRA)

Mineralocorticoid receptor antagonists, such as spironolactone, are commonly used in non-dialysis patients with resistant hypertension. In general, their use is avoided in HD patients because of the potential risk for hyperkalemia. However, two recent trials have reported promising results with spironolactone in dialysis patient. In the Dialysis Outcomes Heart Failure Aldactone Study,^98^ 309 oligoanuric HD patients were randomly assigned to receive 25 mg/day of spironolactone without any restriction on dietary potassium intake (treatment group), and 152 patients were assigned to a control group. During the 3-year follow-up, spironolactone significantly reduced the risks of death from CV events or hospitalization before (HR = 0.40, 95% CI = 0.20–0.81) and after adjustment (HR = 0.38, 95% CI = 0.17–0.83), respectively. The incidence of drug discontinuation due to serious hyperkalemia was 1.9%. In another multicenter RCT, 253 HD or PD patients without heart failure were randomized to 2-year treatment with spironolactone (25 mg/day) or placebo. Add-on MRA therapy reduced the occurrence of the composite primary end point of CV mortality and mitigated the risks for cardiac arrest and sudden death (HR = 0.42, 95% CI = 0.26–0.78), suggesting beneficial effects of low-dose spironolactone on reducing CV morbidity and mortality in dialysis patients.^99^ Importantly, the two aforementioned studies suggest that the mortality reduction with spironolactone exceeds 50% in dialysis patients, which is surprising because few studies have shown such a significant reduction of the mortality rate of dialysis patients.^100^ Indeed, a cardioprotective effect of MRAs in dialysis patients has an established biological basis.^101^ The beneficial effect of MRAs is mediated through improved endothelial function and reduced left ventricular size independent of BP changes, rather than through changes in salt or potassium handling by the kidney. However, the safety of MRAs in this population should be evaluated further. In a recent study of 146 HD patients,^102^ the use of eplerenone (25–50 mg per day) significantly increased the incidence of hyperkalemia (defined as pre-dialysis serum potassium level > 6.5 mmol/L) compared to placebo (RR = 4.50, 95% CI = 14.0–20.2).^102^ Despite the similar rate of permanent drug discontinuation due to hyperkalemia or hypotension (primary end point) between the eplerenone and placebo groups, further adequately powered well-designed studies are required. In addition, which mineralocorticoid receptor antagonist is most suitable for use in ESRD is still questionable. Newer agents, such as finerenone, may have a better safety profile, although this needs further study. The ongoing study Aldosterone Antagonist Chronic Hemodialysis Interventional Survival Trial (ALCHEMIST; NCT01848639) is expected to determine the effectiveness and safety of MRAs in the ESRD patients. Table 3 summarized information for treatment of hypertension in HD patients.

**Resistant hypertension**

Resistant hypertension is defined as uncontrolled hypertension notwithstanding the use of at least three drugs of different classes including diuretics or hypertension controlled with at least four drugs. According to Korean Ambulatory Blood Pressure Monitoring Registry data, the prevalence of resistant hypertension in the general population is about 12%.^103^ In the dialysis population, the prevalence is much higher. European multicenter data with 506 HD patients showed that the prevalence of resistant hypertension with 44 h ABPM criteria (≥130/80 mmHg) was estimated at 25%.^104^ Although fluid overload is a central feature of resistant hypertension in HD patients,^105^ in that study, fluid overload *per se* explains the 33% resistant hypertension and the 67% of patients showed no fluid overload. Non-adherence to medications is another common cause of resistant hypertension.^106^ Chronically non-adherent hypertensive patients who refuse to take medications at home may benefit from the administration of long-acting antihypertensive medications in the dialysis unit. If a treatable cause cannot be found, minoxidil may be effective in reducing BP. The central sympathetic agonists, such as methyldopa and clonidine, are used less frequently because of their adverse effects involving the central nervous system.^107, 108^

Finally, recent RCTs have confirmed the ability of renal denervation to lower BP in patients that are resistant to the BP-lowering effect of multiple antihypertensive drugs. Evidence is limited, however, in patients with ESRD. Renal denervation is an experimental therapy in which sympathetic nerves innervating the kidney are ablated for BP control. The effect of renal denervation was evaluated in a small nonrandomized trial of 24 HD patients who showed resistant hypertension despite maximal medical therapy with confirmed adherence.^109^ The baseline office and 24 h mean SBP in the renal denervation group were 180 ± 112 and 175 ± 11 mmHg, respectively. After renal denervation, an early and persistent reduction of SBP was observed (office SBP: 165 ± 13; 150 ± 7 and 149 ± 11 mmHg; 24 h SBP 163 ± 20, 148 ± 10 and 149 ± 17 mmHg after 1, 6 and 12 months, respectively). The BP-lowering effect was almost always present and statistically significant during both the day and night, suggesting the beneficial role of renal denervation in dialysis patients, too.

**Conclusions**

Hypertension is very common in the dialysis population, but the diagnosis of high BP and optimal treatment target are unclear. At present, however, it is obvious that high BP is associated with increased CV events and the use of anti-hypertensive medications are beneficial for reducing mortality in dialysis patients. Interdialytic BP monitoring, ABPM or home BP monitoring, is superior to the traditional peridialytic BP measurements for predicting long-term outcomes. For treatment of high BP, dietary sodium restriction and maintaining euvolemic status are of paramount importance. Overall, all anti-hypertensive drugs can be used in dialysis population with more recent recommendations of the use of β-blocker as first-line therapy. RCTs with anti-hypertensive drugs selection aiming to reduce mortality are still needed.

**List of abbreviations**

ABPM - Ambulatory blood pressure monitoring

ACEi - Angiotensin-converting enzyme inhibitor

ARB - Angiotensin receptor blocker

BIA - Bioimpedance analysis

BID - Blood Pressure in Dialysis

BP - Blood pressure

CCB - Calcium channel blockers

CI - Confidence interval

CV - Cardiovascular

DBP - Diastolic blood pressure

DW - Dry weight

ESRD - End-stage renal disease

HD - Hemodialysis

HR – Hazard ratio

IQR - interquartile range

KDIGO - Kidney Disease: Improving Global Outcomes

K/DOQI - Kidney Disease Outcomes Quality Initiative

LV - Left ventricle

LVM - Left ventricular mass

LVMI - Left ventricular mass index

MACE - Major adverse cardiovascular event

MRA - Mineralocorticoid receptor antagonist

PD - peritoneal dialysis

PP - pulse pressure

RAS - Renin-angiotensin system

RCT - Randomized controlled trial

RR - Relative risk

SBP - Systolic blood pressure

UF - ultrafiltration

**Declarations**

- **Ethics approval and consent to participate: not applicable**
- **Consent for publication: not applicable**
- **Availability of data and materials: not applicable**
- **Competing interests: The authors declare that they have no competing interests**
- **Funding: none**
- **Authors' contributions: ISK, SMK, and JKK collected data and wrote the paper. THY contributed to the final version of the manuscript.**
- **Acknowledgements:** **The authors thank Medical Illustration & Design, part of the Medical Research Support Services of Yonsei University College of Medicine, for all artistic support related to this work.**

**References**

**1.** Sarafidis PA, Li S, Chen SC, et al. Hypertension awareness, treatment, and control in chronic kidney disease. *Am J Med.* 2008;121(4): 332-340.

**2.** Cocchi R, Degli Esposti E, Fabbri A, et al. Prevalence of hypertension in patients on peritoneal dialysis: results of an Italian multicentre study. *Nephrol Dial Transplant.* 1999;14(6): 1536-1540.

**3.** Menon MK, Naimark DM, Bargman JM, Vas SI, Oreopoulos DG. Long-term blood pressure control in a cohort of peritoneal dialysis patients and its association with residual renal function. *Nephrol Dial Transplant.* 2001;16(11): 2207-2213.

**4.** Kim HC, Lee H, Lee HH, et al. Korea hypertension fact sheet 2021: analysis of nationwide population-based data with special focus on hypertension in women. *Clin Hypertens.* 2022;28(1): 1.

**5.** Agarwal R, Nissenson AR, Batlle D, Coyne DW, Trout JR, Warnock DG. Prevalence, treatment, and control of hypertension in chronic hemodialysis patients in the United States. *Am J Med.* 2003;115(4): 291-297.

**6.** Rocco MV, Yan G, Heyka RJ, Benz R, Cheung AK. Risk factors for hypertension in chronic hemodialysis patients: baseline data from the HEMO study. *Am J Nephrol.* 2001;21(4): 280-288.

**7.** Agarwal R. Epidemiology of interdialytic ambulatory hypertension and the role of volume excess. *Am J Nephrol.* 2011;34(4): 381-390.

**8.** Cheung AK, Rahman M, Reboussin DM, et al. Effects of Intensive BP Control in CKD. *J Am Soc Nephrol.* 2017;28(9): 2812-2823.

**9.** Selby NM, McIntyre CW. The acute cardiac effects of dialysis. *Semin Dial.* 2007;20(3): 220-228.

**10.** Vaios V, Georgianos PI, Liakopoulos V, Agarwal R. Assessment and Management of Hypertension among Patients on Peritoneal Dialysis. *Clin J Am Soc Nephrol.* 2019;14(2): 297-305.

**11.** Slabbert A, Chothia MY. The association between office blood pressure and fluid status using bioimpedance spectroscopy in stable continuous ambulatory peritoneal dialysis patients. *Clin Hypertens.* 2022;28(1): 8.

**12.** K/DOQI clinical practice guidelines on hypertension and antihypertensive agents in chronic kidney disease. *Am J Kidney Dis.* 2004;43(5 Suppl 1): S1-290.

**13.** K/DOQI clinical practice guidelines for cardiovascular disease in dialysis patients. *Am J Kidney Dis.* 2005;45(4 Suppl 3): S1-153.

**14.** Rahman M, Griffin V, Kumar A, Manzoor F, Wright JT, Jr., Smith MC. A comparison of standardized versus "usual" blood pressure measurements in hemodialysis patients. *Am J Kidney Dis.* 2002;39(6): 1226-1230.

**15.** Agarwal R, Peixoto AJ, Santos SF, Zoccali C. Pre- and postdialysis blood pressures are imprecise estimates of interdialytic ambulatory blood pressure. *Clin J Am Soc Nephrol.* 2006;1(3): 389-398.

**16.** Sarafidis PA, Persu A, Agarwal R, et al. Hypertension in dialysis patients: a consensus document by the European Renal and Cardiovascular Medicine (EURECA-m) working group of the European Renal Association-European Dialysis and Transplant Association (ERA-EDTA) and the Hypertension and the Kidney working group of the European Society of Hypertension (ESH). *Nephrol Dial Transplant.* 2017;32(4): 620-640.

**17.** Rohrscheib MR, Myers OB, Servilla KS, et al. Age-related blood pressure patterns and blood pressure variability among hemodialysis patients. *Clin J Am Soc Nephrol.* 2008;3(5): 1407-1414.

**18.** Son HE, Ryu JY, Go S, et al. Association of ambulatory blood pressure monitoring with renal outcome in patients with chronic kidney disease. *Kidney Res Clin Pract.* 2020;39(1): 70-80.

**19.** Sarafidis PA, Persu A, Agarwal R, et al. Hypertension in dialysis patients: a consensus document by the European Renal and Cardiovascular Medicine (EURECA-m) working group of the European Renal Association - European Dialysis and Transplant Association (ERA-EDTA) and the Hypertension and the Kidney working group of the European Society of Hypertension (ESH). *J Hypertens.* 2017;35(4): 657-676.

**20.** Agarwal R. Blood pressure and mortality among hemodialysis patients. *Hypertension.* 2010;55(3): 762-768.

**21.** Alborzi P, Patel N, Agarwal R. Home blood pressures are of greater prognostic value than hemodialysis unit recordings. *Clin J Am Soc Nephrol.* 2007;2(6): 1228-1234.

**22.** Agarwal R, Andersen MJ, Bishu K, Saha C. Home blood pressure monitoring improves the diagnosis of hypertension in hemodialysis patients. *Kidney Int.* 2006;69(5): 900-906.

**23.** Bansal N, McCulloch CE, Rahman M, et al. Blood pressure and risk of all-cause mortality in advanced chronic kidney disease and hemodialysis: the chronic renal insufficiency cohort study. *Hypertension.* 2015;65(1): 93-100.

**24.** Agarwal R, Metiku T, Tegegne GG, et al. Diagnosing hypertension by intradialytic blood pressure recordings. *Clin J Am Soc Nephrol.* 2008;3(5): 1364-1372.

**25.** Jung JY, Yoo KD, Kang E, et al. Korean Society of Nephrology 2021 Clinical Practice Guideline for Optimal Hemodialysis Treatment. *Kidney Res Clin Pract.* 2021;40(Suppl 1): S1-s37.

**26.** Camafort M, Redón J, Pyun WB, Coca A. Intensive blood pressure lowering: a practical review. *Clin Hypertens.* 2020;26(1): 21.

**27.** Zager PG, Nikolic J, Brown RH, et al. "U" curve association of blood pressure and mortality in hemodialysis patients. Medical Directors of Dialysis Clinic, Inc. *Kidney Int.* 1998;54(2): 561-569.

**28.** Hannedouche T, Roth H, Krummel T, et al. Multiphasic effects of blood pressure on survival in hemodialysis patients. *Kidney Int.* 2016;90(3): 674-684.

**29.** Miskulin DC, Gassman J, Schrader R, et al. BP in Dialysis: Results of a Pilot Study. *J Am Soc Nephrol.* 2018;29(1): 307-316.

**30.** Flythe JE, Xue H, Lynch KE, Curhan GC, Brunelli SM. Association of mortality risk with various definitions of intradialytic hypotension. *J Am Soc Nephrol.* 2015;26(3): 724-734.

**31.** Daugirdas JT. Measuring intradialytic hypotension to improve quality of care. *J Am Soc Nephrol.* 2015;26(3): 512-514.

**32.** Sands JJ, Usvyat LA, Sullivan T, et al. Intradialytic hypotension: frequency, sources of variation and correlation with clinical outcome. *Hemodial Int.* 2014;18(2): 415-422.

**33.** Stefánsson BV, Brunelli SM, Cabrera C, et al. Intradialytic hypotension and risk of cardiovascular disease. *Clin J Am Soc Nephrol.* 2014;9(12): 2124-2132.

**34.** Chang TI, Paik J, Greene T, et al. Intradialytic hypotension and vascular access thrombosis. *J Am Soc Nephrol.* 2011;22(8): 1526-1533.

**35.** Mazzuchi N, Carbonell E, Fernández-Cean J. Importance of blood pressure control in hemodialysis patient survival. *Kidney Int.* 2000;58(5): 2147-2154.

**36.** Kalantar-Zadeh K, Kilpatrick RD, McAllister CJ, Greenland S, Kopple JD. Reverse epidemiology of hypertension and cardiovascular death in the hemodialysis population: the 58th annual fall conference and scientific sessions. *Hypertension.* 2005;45(4): 811-817.

**37.** Robinson BM, Tong L, Zhang J, et al. Blood pressure levels and mortality risk among hemodialysis patients in the Dialysis Outcomes and Practice Patterns Study. *Kidney Int.* 2012;82(5): 570-580.

**38.** Tentori F, Hunt WC, Rohrscheib M, et al. Which targets in clinical practice guidelines are associated with improved survival in a large dialysis organization? *J Am Soc Nephrol.* 2007;18(8): 2377-2384.

**39.** Li Z, Lacson E, Jr., Lowrie EG, et al. The epidemiology of systolic blood pressure and death risk in hemodialysis patients. *Am J Kidney Dis.* 2006;48(4): 606-615.

**40.** Suzuki H, Kanno Y, Sugahara S, et al. Effect of angiotensin receptor blockers on cardiovascular events in patients undergoing hemodialysis: an open-label randomized controlled trial. *Am J Kidney Dis.* 2008;52(3): 501-506.

**41.** Takeda A, Toda T, Fujii T, Shinohara S, Sasaki S, Matsui N. Discordance of influence of hypertension on mortality and cardiovascular risk in hemodialysis patients. *Am J Kidney Dis.* 2005;45(1): 112-118.

**42.** Heerspink HJ, Ninomiya T, Zoungas S, et al. Effect of lowering blood pressure on cardiovascular events and mortality in patients on dialysis: a systematic review and meta-analysis of randomised controlled trials. *Lancet.* 2009;373(9668): 1009-1015.

**43.** Agarwal R, Sinha AD. Cardiovascular protection with antihypertensive drugs in dialysis patients: systematic review and meta-analysis. *Hypertension.* 2009;53(5): 860-866.

**44.** Cice G, Ferrara L, D'Andrea A, et al. Carvedilol increases two-year survivalin dialysis patients with dilated cardiomyopathy: a prospective, placebo-controlled trial. *J Am Coll Cardiol.* 2003;41(9): 1438-1444.

**45.** Zannad F, Kessler M, Lehert P, et al. Prevention of cardiovascular events in end-stage renal disease: results of a randomized trial of fosinopril and implications for future studies. *Kidney Int.* 2006;70(7): 1318-1324.

**46.** Takahashi A, Takase H, Toriyama T, et al. Candesartan, an angiotensin II type-1 receptor blocker, reduces cardiovascular events in patients on chronic haemodialysis--a randomized study. *Nephrol Dial Transplant.* 2006;21(9): 2507-2512.

**47.** Tepel M, Hopfenmueller W, Scholze A, Maier A, Zidek W. Effect of amlodipine on cardiovascular events in hypertensive haemodialysis patients. *Nephrol Dial Transplant.* 2008;23(11): 3605-3612.

**48.** Amar J, Vernier I, Rossignol E, et al. Nocturnal blood pressure and 24-hour pulse pressure are potent indicators of mortality in hemodialysis patients. *Kidney Int.* 2000;57(6): 2485-2491.

**49.** Klassen PS, Lowrie EG, Reddan DN, et al. Association between pulse pressure and mortality in patients undergoing maintenance hemodialysis. *Jama.* 2002;287(12): 1548-1555.

**50.** Foley RN, Herzog CA, Collins AJ. Blood pressure and long-term mortality in United States hemodialysis patients: USRDS Waves 3 and 4 Study. *Kidney Int.* 2002;62(5): 1784-1790.

**51.** Stidley CA, Hunt WC, Tentori F, et al. Changing relationship of blood pressure with mortality over time among hemodialysis patients. *J Am Soc Nephrol.* 2006;17(2): 513-520.

**52.** Wright JT, Jr., Williamson JD, Whelton PK, et al. A Randomized Trial of Intensive versus Standard Blood-Pressure Control. *N Engl J Med.* 2015;373(22): 2103-2116.

**53.** Flythe JE, Chang TI, Gallagher MP, et al. Blood pressure and volume management in dialysis: conclusions from a Kidney Disease: Improving Global Outcomes (KDIGO) Controversies Conference. *Kidney Int.* 2020;97(5): 861-876.

**54.** Cheung AK, Chang TI, Cushman WC, et al. Executive summary of the KDIGO 2021 Clinical Practice Guideline for the Management of Blood Pressure in Chronic Kidney Disease. *Kidney Int.* 2021;99(3): 559-569.

**55.** Jindal K, Chan CT, Deziel C, et al. Hemodialysis clinical practice guidelines for the Canadian Society of Nephrology. *J Am Soc Nephrol.* 2006;17(3 Suppl 1): S1-27.

**56.** Hirakata H, Nitta K, Inaba M, et al. Japanese Society for Dialysis Therapy guidelines for management of cardiovascular diseases in patients on chronic hemodialysis. *Ther Apher Dial.* 2012;16(5): 387-435.

**57.** Kooman J, Basci A, Pizzarelli F, et al. EBPG guideline on haemodynamic instability. *Nephrol Dial Transplant.* 2007;22 Suppl 2: ii22-44.

**58.** Inrig JK, Patel UD, Toto RD, Szczech LA. Association of blood pressure increases during hemodialysis with 2-year mortality in incident hemodialysis patients: a secondary analysis of the Dialysis Morbidity and Mortality Wave 2 Study. *Am J Kidney Dis.* 2009;54(5): 881-890.

**59.** Agarwal R, Light RP. Intradialytic hypertension is a marker of volume excess. *Nephrol Dial Transplant.* 2010;25(10): 3355-3361.

**60.** Van Buren PN, Kim C, Toto R, Inrig JK. Intradialytic hypertension and the association with interdialytic ambulatory blood pressure. *Clin J Am Soc Nephrol.* 2011;6(7): 1684-1691.

**61.** Udayaraj UP, Steenkamp R, Caskey FJ, et al. Blood pressure and mortality risk on peritoneal dialysis. *Am J Kidney Dis.* 2009;53(1): 70-78.

**62.** Van Biesen W, Williams JD, Covic AC, et al. Fluid status in peritoneal dialysis patients: the European Body Composition Monitoring (EuroBCM) study cohort. *PLoS One.* 2011;6(2): e17148.

**63.** Ronco C, Verger C, Crepaldi C, et al. Baseline hydration status in incident peritoneal dialysis patients: the initiative of patient outcomes in dialysis (IPOD-PD study)†. *Nephrol Dial Transplant.* 2015;30(5): 849-858.

**64.** Tangwonglert T, Davenport A. Changes in extracellular water and left ventricular mass in peritoneal dialysis patients. *Kidney Res Clin Pract.* 2021;40(1): 135-142.

**65.** Wang AY, Brimble KS, Brunier G, et al. ISPD Cardiovascular and Metabolic Guidelines in Adult Peritoneal Dialysis Patients Part I - Assessment and Management of Various Cardiovascular Risk Factors. *Perit Dial Int.* 2015;35(4): 379-387.

**66.** Alexandrou ME, Loutradis C, Schoina M, et al. Ambulatory blood pressure profile and blood pressure variability in peritoneal dialysis compared with hemodialysis and chronic kidney disease patients. *Hypertens Res.* 2020;43(9): 903-913.

**67.** Liu JH, Chen CC, Wang SM, et al. Association between pulse pressure and 30-month all-cause mortality in peritoneal dialysis patients. *Am J Hypertens.* 2008;21(12): 1318-1323.

**68.** Fang W, Yang X, Bargman JM, Oreopoulos DG. Association between pulse pressure and mortality in patients undergoing peritoneal dialysis. *Perit Dial Int.* 2009;29(2): 163-170.

**69.** Afshinnia F, Zaky ZS, Metireddy M, Segal JH. Reverse Epidemiology of Blood Pressure in Peritoneal Dialysis Associated with Dynamic Deterioration of Left Ventricular Function. *Perit Dial Int.* 2016;36(2): 154-162.

**70.** Goldfarb-Rumyantzev AS, Baird BC, Leypoldt JK, Cheung AK. The association between BP and mortality in patients on chronic peritoneal dialysis. *Nephrol Dial Transplant.* 2005;20(8): 1693-1701.

**71.** Shin J, Lee CH. The roles of sodium and volume overload on hypertension in chronic kidney disease. *Kidney Res Clin Pract.* 2021;40(4): 542-554.

**72.** Sinha AD, Agarwal R. Can chronic volume overload be recognized and prevented in hemodialysis patients? The pitfalls of the clinical examination in assessing volume status. *Semin Dial.* 2009;22(5): 480-482.

**73.** Nongnuch A, Campbell N, Stern E, El-Kateb S, Fuentes L, Davenport A. Increased postdialysis systolic blood pressure is associated with extracellular overhydration in hemodialysis outpatients. *Kidney Int.* 2015;87(2): 452-457.

**74.** Günal AI, Duman S, Ozkahya M, et al. Strict volume control normalizes hypertension in peritoneal dialysis patients. *Am J Kidney Dis.* 2001;37(3): 588-593.

**75.** Abu-Alfa AK, Burkart J, Piraino B, Pulliam J, Mujais S. Approach to fluid management in peritoneal dialysis: a practical algorithm. *Kidney Int Suppl.* 2002(81): S8-16.

**76.** Agarwal R, Alborzi P, Satyan S, Light RP. Dry-weight reduction in hypertensive hemodialysis patients (DRIP): a randomized, controlled trial. *Hypertension.* 2009;53(3): 500-507.

**77.** Frame AA, Wainford RD. Renal sodium handling and sodium sensitivity. *Kidney Res Clin Pract.* 2017;36(2): 117-131.

**78.** Kooman JP, van der Sande F, Leunissen K, Locatelli F. Sodium balance in hemodialysis therapy. *Semin Dial.* 2003;16(5): 351-355.

**79.** Tomson CR. Advising dialysis patients to restrict fluid intake without restricting sodium intake is not based on evidence and is a waste of time. *Nephrol Dial Transplant.* 2001;16(8): 1538-1542.

**80.** Davies S, Carlsson O, Simonsen O, et al. The effects of low-sodium peritoneal dialysis fluids on blood pressure, thirst and volume status. *Nephrol Dial Transplant.* 2009;24(5): 1609-1617.

**81.** Kim KI, Ihm SH, Kim GH, et al. 2018 Korean society of hypertension guidelines for the management of hypertension: part III-hypertension in special situations. *Clin Hypertens.* 2019;25: 19.

**82.** Mancia G, Fagard R, Narkiewicz K, et al. 2013 ESH/ESC Guidelines for the management of arterial hypertension: the Task Force for the management of arterial hypertension of the European Society of Hypertension (ESH) and of the European Society of Cardiology (ESC). *J Hypertens.* 2013;31(7): 1281-1357.

**83.** Ruggenenti P, Podestà MA, Trillini M, et al. Ramipril and Cardiovascular Outcomes in Patients on Maintenance Hemodialysis: The ARCADIA Multicenter Randomized Controlled Trial. *Clin J Am Soc Nephrol.* 2021;16(4): 575-587.

**84.** Peters CD, Kjaergaard KD, Jensen JD, et al. No significant effect of angiotensin II receptor blockade on intermediate cardiovascular end points in hemodialysis patients. *Kidney Int.* 2014;86(3): 625-637.

**85.** Iseki K, Arima H, Kohagura K, et al. Effects of angiotensin receptor blockade (ARB) on mortality and cardiovascular outcomes in patients with long-term haemodialysis: a randomized controlled trial. *Nephrol Dial Transplant.* 2013;28(6): 1579-1589.

**86.** Tai DJ, Lim TW, James MT, Manns BJ, Tonelli M, Hemmelgarn BR. Cardiovascular effects of angiotensin converting enzyme inhibition or angiotensin receptor blockade in hemodialysis: a meta-analysis. *Clin J Am Soc Nephrol.* 2010;5(4): 623-630.

**87.** Zoccali C, Mallamaci F. Pleiotropic effects of angiotensin II blockers in hemodialysis patients: myth or reality? *Kidney Int.* 2014;86(3): 469-471.

**88.** Agarwal R, Sinha AD, Pappas MK, Abraham TN, Tegegne GG. Hypertension in hemodialysis patients treated with atenolol or lisinopril: a randomized controlled trial. *Nephrol Dial Transplant.* 2014;29(3): 672-681.

**89.** Zoccali C, Mallamaci F, Parlongo S, et al. Plasma norepinephrine predicts survival and incident cardiovascular events in patients with end-stage renal disease. *Circulation.* 2002;105(11): 1354-1359.

**90.** Denker MG, Cohen DL. Antihypertensive Medications in End-Stage Renal Disease. *Semin Dial.* 2015;28(4): 330-336.

**91.** Jadoul M, Thumma J, Fuller DS, et al. Modifiable practices associated with sudden death among hemodialysis patients in the Dialysis Outcomes and Practice Patterns Study. *Clin J Am Soc Nephrol.* 2012;7(5): 765-774.

**92.** Weir MA, Dixon SN, Fleet JL, et al. β-Blocker dialyzability and mortality in older patients receiving hemodialysis. *J Am Soc Nephrol.* 2015;26(4): 987-996.

**93.** Wu PH, Lin YT, Kuo MC, et al. β-blocker dialyzability and the risk of mortality and cardiovascular events in patients undergoing hemodialysis. *Nephrol Dial Transplant.* 2020;35(11): 1959-1965.

**94.** Tella A, Vang W, Ikeri E, et al. β-Blocker Use and Cardiovascular Outcomes in Hemodialysis: A Systematic Review. *Kidney Med.* 2022;4(5): 100460.

**95.** London GM, Marchais SJ, Guerin AP, et al. Salt and water retention and calcium blockade in uremia. *Circulation.* 1990;82(1): 105-113.

**96.** Aslam S, Santha T, Leone A, Wilcox C. Effects of amlodipine and valsartan on oxidative stress and plasma methylarginines in end-stage renal disease patients on hemodialysis. *Kidney Int.* 2006;70(12): 2109-2115.

**97.** Pantosti A, Boccia D, D'Ambrosio F, Recchia S, Orefici G, Moro ML. Inferring the potential success of pneumococcal vaccination in Italy: serotypes and antibiotic resistance of Streptococcus pneumoniae isolates from invasive diseases. *Microb Drug Resist.* 2003;9 Suppl 1: S61-68.

**98.** Matsumoto Y, Mori Y, Kageyama S, et al. Spironolactone reduces cardiovascular and cerebrovascular morbidity and mortality in hemodialysis patients. *J Am Coll Cardiol.* 2014;63(6): 528-536.

**99.** Lin C, Zhang Q, Zhang H, Lin A. Long-Term Effects of Low-Dose Spironolactone on Chronic Dialysis Patients: A Randomized Placebo-Controlled Study. *J Clin Hypertens (Greenwich).* 2016;18(2): 121-128.

**100.** Kramann R, Floege J, Ketteler M, Marx N, Brandenburg VM. Medical options to fight mortality in end-stage renal disease: a review of the literature. *Nephrol Dial Transplant.* 2012;27(12): 4298-4307.

**101.** Agarwal A, Cheung AK. Mineralocorticoid Receptor Antagonists in ESKD. *Clin J Am Soc Nephrol.* 2020;15(7): 1047-1049.

**102.** Walsh M, Manns B, Garg AX, et al. The Safety of Eplerenone in Hemodialysis Patients: A Noninferiority Randomized Controlled Trial. *Clin J Am Soc Nephrol.* 2015;10(9): 1602-1608.

**103.** Choi SI, Kim SK, Park S, et al. Prevalence of resistant hypertension and associated factors for blood pressure control status with optimal medical therapy using Korean ambulatory blood pressure monitoring registry data. *Clin Hypertens.* 2015;22: 8.

**104.** Mallamaci F, Torino C, Sarafidis P, et al. Treatment-resistant hypertension in the hemodialysis population: a 44-h ambulatory blood pressure monitoring-based study. *J Hypertens.* 2020;38(9): 1849-1856.

**105.** Zoccali C, Moissl U, Chazot C, et al. Chronic Fluid Overload and Mortality in ESRD. *J Am Soc Nephrol.* 2017;28(8): 2491-2497.

**106.** Mechta Nielsen T, Frøjk Juhl M, Feldt-Rasmussen B, Thomsen T. Adherence to medication in patients with chronic kidney disease: a systematic review of qualitative research. *Clin Kidney J.* 2018;11(4): 513-527.

**107.** Ross EA, Pittman TB, Koo LC. Strategy for the treatment of noncompliant hypertensive hemodialysis patients. *Int J Artif Organs.* 2002;25(11): 1061-1065.

**108.** Hörl MP, Hörl WH. Drug therapy for hypertension in hemodialysis patients. *Semin Dial.* 2004;17(4): 288-294.

**109.** Scalise F, Sole A, Singh G, et al. Renal denervation in patients with end-stage renal disease and resistant hypertension on long-term haemodialysis. *J Hypertens.* 2020;38(5): 936-942.

**Figure legend**

**Fig.1 Summary of general management strategy for patients with hemodialysis (HD) or peritoneal dialysis (PD).**

**Table 1. Summary of diagnosis of hypertension in HD patients**

| - ABPM is the gold standard for the diagnosis of hypertension in HD patients. If ABPM is not available, home BP recordings can be a good alternative for accurate BP measurements. |
| --- |
| - An average home BP ≥ 135/85 mmHg or ambulatory BP ≥ 130/80 mmHg is regarded as high BP in HD patients, and in general, the target of self-measured home BP is less than 130/80 mmHg. |
| - When neither ABPM nor home BP measurements are available, in-office BP measurements outside of the dialysis unit or median midweek peridialytic BP may be acceptable. |
| - Increased pulse pressure/arterial stiffness can be another determinant for predicting adverse CV outcomes. |
| - There might be a time-varying effect between high BP and mortality, suggesting the need for long-term follow-up data for predict mortality. |
| - Intradialytic BP pattern should also be considered to avoid serious complications of HD. |

**Table 2. Summary of diagnosis of hypertension in PD patients**

| - Chronic subclinical hypervolemia is very common in PD patients. For PD patients with high BP, an assessment of volume status should be a priority. |
| --- |
| - ABPM is the gold standard for the diagnosis of hypertension in PD patients. However, data assessing the validity of peridialytic, office, and home BP are limited in PD patients. |
| - An average home BP ≥ 135/85 mmHg or ambulatory BP ≥ 130/80 mmHg is regarded as high BP in PD patients, too. |
| - Similar to the HD patients, there may be an effect of dialysis vintage on the relationship between high BP and long-term mortality in PD patients. |

**Table 3 Summary of treatment of hypertension in dialysis patients**

| - Patient heterogeneity and scarcity of comparative evidence preclude recommending any medication class over the other for all dialysis patients. |
| --- |
| - Most ARBs are not dialyzed during conventional dialysis and may be used for sustained BP reduction. However, RCTs have failed to confirm the benefits of RASi in dialysis patients as in the general population. |
| - β-blockers can be used as the first-line therapy in dialysis patients since they can control the sympathetic overactivity and LVH which contribute to the high incidence of arrhythmias and sudden cardiac death. |
| - In a recent study, the use of mineralocorticoid receptor antagonists such as spironolactone showed promising results in reducing mortality by more than 50% in dialysis patients. However, the safety issue such as hyperkalemia or hypotension should be evaluated further. |
| - Volume overload or nonadherence to medications are common causes of resistant hypertension in dialysis patients. |
